# Optimal combinations of control strategies and cost-effective analysis for visceral leishmaniasis disease transmission

**DOI:** 10.1371/journal.pone.0172465

**Published:** 2017-02-21

**Authors:** Santanu Biswas, Abhishek Subramanian, Ibrahim M. ELMojtaba, Joydev Chattopadhyay, Ram Rup Sarkar

**Affiliations:** 1 Department of Mathematics, Adamas University, Barasat, Kolkata, India; 2 Chemical Engineering and Process Development, CSIR-National Chemical Laboratory, Dr. Homi Bhabha Road, Pune, 411008, India; 3 Academy of Scientific & Innovative Research (AcSIR), CSIR-NCL Campus, Pune - 411008, India; 4 Department of Mathematics and Statistics, College of Science - Sultan Qaboos University, P.O. box - 36, Muscat, Oman; 5 Agricultural and Ecological Research Unit, Indian Statistical Institute, 203, B. T. Road, Kolkata, 700108, India; Academic Medical Centre, NETHERLANDS

## Abstract

Visceral leishmaniasis (VL) is a deadly neglected tropical disease that poses a serious problem in various countries all over the world. Implementation of various intervention strategies fail in controlling the spread of this disease due to issues of parasite drug resistance and resistance of sandfly vectors to insecticide sprays. Due to this, policy makers need to develop novel strategies or resort to a combination of multiple intervention strategies to control the spread of the disease. To address this issue, we propose an extensive SIR-type model for anthroponotic visceral leishmaniasis transmission with seasonal fluctuations modeled in the form of periodic sandfly biting rate. Fitting the model for real data reported in South Sudan, we estimate the model parameters and compare the model predictions with known VL cases. Using optimal control theory, we study the effects of popular control strategies namely, drug-based treatment of symptomatic and PKDL-infected individuals, insecticide treated bednets and spray of insecticides on the dynamics of infected human and vector populations. We propose that the strategies remain ineffective in curbing the disease individually, as opposed to the use of optimal combinations of the mentioned strategies. Testing the model for different optimal combinations while considering periodic seasonal fluctuations, we find that the optimal combination of treatment of individuals and insecticide sprays perform well in controlling the disease for the time period of intervention introduced. Performing a cost-effective analysis we identify that the same strategy also proves to be efficacious and cost-effective. Finally, we suggest that our model would be helpful for policy makers to predict the best intervention strategies for specific time periods and their appropriate implementation for elimination of visceral leishmaniasis.

## Introduction

Leishmaniasis is the world’s second largest parasitic killer and is caused by protozoan parasites belonging to the *Leishmania* genus. There are four different clinical manifestations of the disease − visceral leishmaniasis (VL), the post-kala-azar dermal leishmaniasis (PKDL), cutaneous leishmaniasis (CL) and cutaneous leishmaniasis with involvement of lesions of the mucous membranes, which is also called mucocutaneous leishmaniasis (MCL). Visceral leishmaniasis (VL), sometimes usually referred to as “kala-azar” (KA) is the deadliest form of leishmaniasis and is the causal reason for about 20,000 to 40,000 deaths worldwide, with total reported VL cases between 200,000 to 400,000 [1]. Visceral leishmaniasis (VL) has been targeted by the WHO for elimination as it is fatal, if left untreated. According to WHO, most of the cases of VL have been reported in the Indian subcontinent, Sudan, Ethiopia, and Brazil [2].

Apart from the heterogeneity in the clinical manifestations, the problem is further complicated by the presence of “asymptomatic” infections where the patients do not display any symptoms of the disease [3] and hence, their detection poses a challenge. In addition, some VL treated patients (6 months to several years after the treatment regimen) show a macular, maculopapular, and nodular rash that contain dormant parasites [2, 4, 5]. These individuals are themselves recovered, but serve as an active source of new infection when exposed to vectors. Such individuals are referred to as post-kala-azar dermal leishmaniasis (PKDL) infected.

The severity of this disease at such different scales thus, demands for efficient implementation of disease intervention strategies that can limit the spread of the infection among human populations. Recent studies on VL intervention strategies can be broadly classified under four groups; studies relating to—animal reservoir control, vector population control, human reservoir control and finally a group that includes studies where multiple interventions were conducted concurrently [6]. Animal reservoirs are eliminated from infection spread by either culling them [7], or use of canine vaccines [8] and insecticide impregnated collars [9]. The sandfly vector populations are controlled by spray of insecticides [10, 11] and use of treated bednets [12]. As part of human reservoir control, drug-based treatment of infected individuals is one of the large scale programmes undertaken by WHO to reduce the incidence of VL cases in highly concentrated areas [13].

Although these methods are the most commonly used, there are problems and issues with the use of each aforementioned strategy. Lack of recording the actual number of reservoirs in a given area poses a major challenge for animal intervention strategies [14, 15]. An important problem of insecticide sprays or insecticide treated bednets is the increasing resistance of sandflies to insecticides like DDT and deltamethrin [16, 17]. Similarly, continued treatment of infected individuals with antimonials and miltefosine lead to drug resistance within the parasites, where patients stop responding to drug treatments [18, 19]. The issues of implementation of strategies, and the associated problems with each of these strategies, further emphasizes the requirement of novel intervention strategies or the use of effective combinations of the existing strategies to target the elimination of the disease.

Mathematical models provide a detailed framework to study, analyze the dynamics of VL disease transmission and further contribute towards optimal choice of intervention strategies to control VL spread [3]. Historically, epidemiological models have long been proposed to understand leishmaniasis disease transmission and its control. The inter-epidemic periods between 1875 and 1950 in Assam, India was studied by Dye [20] using a deterministic model to describe the dynamics of VL. This model was extended to canine VL in Malta [21] to explain the efficacy of various control methods [22]. After this pioneering work, many mathematical models have been employed to understand the VL transmission dynamics [1, 23–30] but only few articles attempt to describe the detailed VL disease transmission dynamics [1, 25–30]. Transmission of VL can also affect animals. *Leishmania donovani* has been found in animals in East Africa; in Brazil, high levels of infection occur in dog populations (canine or CVL) [3]. Through anthroponotic medium or the zoonotic medium; visceral leishmaniasis can be transmitted between human to human or between animal and human respectively [5] via sandflies [31]. Burattini et al. [32], developed an SEIR type model between sandfly, animal and human populations for zoonotic transmission of visceral leishmaniasis. Following up, many other models [1, 25, 28–30, 33, 34] consider zoonotic transmission along with potential PKDL progression rate in humans via the addition of another infective stage. It is important to note here that most of these studies were dedicated to understand the visceral leishmaniasis transmission and do not directly focus on control of disease spread. The model by Stauch et al. [28] predicts the role of long-term intervention strategies, combined with active case detection and include efficacious treatment. Later ELmojtaba et al. [34] also, formulated a compartmental model and applied two optimal controls, namely treatment and vaccination within the model to investigate optimal strategies for controlling the spread of the disease. A recent mathematical model [35] attempted to understand the effect of specific optimal strategies for controlling anthroponotic cutaneous leishmaniasis in human populations; although this model only focuses on individual control strategies which are ineffective in controlling disease spread and is also not validated with real population data.

With this background, we modify the model of ELmojtaba et al. [25] and propose a detailed compartment-based mathematical model to explain the anthroponotic VL transmission dynamics in three distinct populations—the human and the animal as hosts, and sandfly as the vector, with the detailed analysis of the human infected population into its clinically distinct classes namely, asymptomatic, symptomatic, and PKDL infected. Adding to the complex nature of transmission, the disease also can have a seasonal fluctuation depending on the vector population [31]. To capture the presence of possible seasonal pattern observed within the data of reported VL cases, we include a periodicity factor in our model. We analyze this model to derive conditions for positive invariance, global stability of the unique disease free equilibrium, expression for the basic reproduction number in a periodic environment, and the conditions for the existence and permanence of at least one positive endemic periodic solution. We attempt to fit the model outcomes to the number of new VL cases occurred in South Sudan to estimate model parameters. We also perform parameter sensitivity analysis to identify the most sensitive parameters in our model.

Although the aforementioned studies do propose intervention techniques for disease control; none of the studies previously investigate the effects of the interventions in the disease dynamics while accounting for periodic seasonal fluctuations, their efficacy and cost-effectiveness which may sometimes be limited by availability of resources. We further propose combinations of known preventive measures, namely, (i) treated bednets, (ii) treatment of infective humans and (iii) spray of insecticides and compare them with each other to understand the short and long term effects of the interventions on infected vector and human populations. We also use optimal control theory to study the efficacy of different strategic combinations of VL interventions in disease elimination as well as their cost-effectiveness. We explore the effects of proposed combinations and calculate the Infection Averted Ratio (IAR) with the Incremental Cost-Effectiveness Ratio (ICER) to investigate the efficacy of the combinations to eliminate disease from the population and their cost-effectiveness. Specifically, we emphasize that, by carrying out such a comparative analysis; predicting the involved costs and the corresponding outcomes of alternative control strategies can be useful to decision makers, who are often faced by the challenge of resource allocation. An optimal balance among different types of interventions may significantly reduce the number of VL cases and deaths at a minimal cost. This can lead to a well-coordinated effort and an effective implementation of strategies for disease control.

## Methods

### Model construction

The transmission dynamics of VL in the Indian subcontinent was modeled by a system of ordinary differential equations as per [1, 28, 29]. Following this framework, we consider the basic SIR type model with respect to history of infection within the human population. The model comprises of the human, reservoir and sandfly populations with seasonally forced biting rates on the sandfly population. The human population *N*_*H*_(*t*) is divided into six subpopulations namely susceptible *S*_*H*_, asymptomatically infected *I*_*A*_, symptomatic infected *I*_*H*_, transient *T*_*H*_, PKDL-infected *P*_*H*_ and recovered *R*_*H*_ individuals.

$$N_{H}(t)=S_{H}(t)+I_{A}(t)+I_{H}(t)+T_{H}(t)+P_{H}(t)+R_{H}(t)$$

Similarly, let the reservoir host population be divided into two categories, susceptible reservoir, *S*_*R*_(*t*), and infected reservoir, *I*_*R*_(*t*), such that

$$N_{R}\left(t\right)=S_{R}\left(t\right)+I_{R}\left(t\right)$$

The total vector (sandfly) population, denoted by *N*_*V*_ is subdivided into susceptible sandflies *S*_*V*_(*t*), and infected sandflies *I*_*V*_(*t*), such that

$$N_{V}\left(t\right)=S_{V}\left(t\right)+I_{V}\left(t\right).$$

All the humans initially remain susceptible to infection and are assumed to grow in number with a constant birth Λ_*H*_ and death *μ*_*h*_ rate. After being bitten by an infectious sandfly, susceptible humans (*S*_*H*_) are considered to become asymptomatically infected (*I*_*A*_) with force of infection $ab\frac{I_{V}}{N_{H}}$ where *a* is the mean rate of bites per sandfly and b is the sandfly to human (reservoir) transmission probability. The per-capita biting rate of sandflies *a* is equal to the number of bites received per human from sandfly due to conservation of bites mechanism. Asymptomatic stages can include those humans with sub-symptomatic and non symptomatic early infection. Here, the asymptomatic stage (*I*_*A*_) describes the subset of all those humans capable of contributing towards disease transmission. If alive, they remain asymptomatic for $\frac{1}{\gamma_{h}}$ days. A fraction of these individuals (*ρ*_1_) develop symptomatic KA (*I*_*H*_), some (*ρ*_2_) become PKDL-infected (*P*_*H*_) and the remaining *ρ*_3_ = 1 − *ρ*_1_ − *ρ*_2_ recover from the asymptomatic infected stage (*I*_*A*_). Symptomatic humans (*I*_*H*_) are eligible for treatment. These individuals die due to VL at an average rate *δ*, or get treated at an average rate *α*_1_. A proportion of patients *σ*, from *I*_*H*_ stage successfully combat the parasites and recover from the infection (*R*_*H*_). The remaining proportion (1 − *σ*) of patients putatively enter into the dormant stage (*T*_*H*_); followed by a PKDL (*P*_*H*_) infection if on an average they survive upto $\frac{1}{\delta_{p}}$ days [3]. Humans with PKDL get treated at an average rate *α*_2_, or recover naturally at an average rate *β*. Cellular immunity remains for $\frac{1}{p_{r}}$ days, after which recovered humans (*R*_*H*_) again become susceptible (*S*_*H*_).

Susceptible reservoirs are recruited from the population at a constant rate Λ_*R*_, and acquire infection VL following contacts with infected sandflies at a rate $ab\frac{I_{V}}{N_{H}}$ where *a* and *b* as described above. We assume that the transmission probability per bite is the same for human and reservoir because sandflies do not distinguish between humans and reservoirs. It is also assumed that reservoirs do not die due to the disease, but are limited by a per capita natural mortality rate *μ*_*r*_.

Susceptible sandflies are recruited at a constant rate Λ_*V*_, and acquire VL infection following contacts with human infected with visceral leishmaniasis(*I*_*A*_ and *I*_*H*_) or human having PKDL (*P*_*H*_) or reservoir infected (*I*_*R*_) with visceral leishmaniasis at an average rate equal to $\mu_{1}ac\frac{I_{A}}{N_{H}}+ac\frac{I_{H}}{N_{H}}+ac\frac{P_{H}}{N_{H}}+\mu_{2}ac\frac{I_{R}}{N_{R}}$, where *a* is the per-capita biting rate, and *c* is the transmission probability for sandfly infection. The infection probabilities of sandfly depend on the stage of infection [28]. We assume that *μ*_1_ and *μ*_2_ are the respective infection probabilities of sandfly for biting humans and reservoir in the stages *I*_*A*_, *I*_*H*_, *P*_*H*_, *I*_*R*_. Sandflies suffer natural mortality at a per-capita rate *μ*_*v*_ regardless of their infection status.

Abubakar et. al. [36] described that, the monthly distribution of VL cases reflected the general pattern observed in South Sudan, with less cases during the transmission season (April to June) and a peak during the dry season, beginning continuously in September. Hence, we assumed a periodic form for the biting rate as follows: $a\left(t\right)=a_{0}\left(1+\delta_{r}\sin\frac{2\pi t}{12}\right)$. The biting rate *a*(*t*) of the sandfly population varies periodically with different temperatures which is assumed to be time periodic for a period of 12 months. *a*_0_ denote the average biting rate and *δ*_*r*_ denotes the amplitude of seasonality [37–40].

With this assumption and the description of the terms, we get the following system of differential equations:

$$\begin{array}{ccc} S_{H}^{\prime } & = & \Lambda_{H}-a\left(t\right)bI_{V}\frac{S_{H}}{N_{H}}-\mu_{H}S_{H}+\rho_{r}R_{H}\\ I_{A}^{\prime } & = & a\left(t\right)bI_{V}\frac{S_{H}}{N_{H}}-\left(\gamma_{H}+\mu_{H}\right)I_{A}\\ I_{H}^{\prime } & = & \rho_{1}\gamma_{H}I_{A}-\left(\alpha_{1}+\delta +\mu_{H}\right)I_{H}\\ T_{H}^{\prime } & = & \left(1-\sigma \right)\alpha_{1}I_{H}-\left(\delta_{p}+\mu_{H}\right)T_{H}\\ P_{H}^{\prime } & = & \rho_{2}\gamma_{h}I_{A}+\delta_{p}T_{H}-\left(\alpha_{2}+\beta +\mu_{H}\right)P_{H}\\ R_{H}^{\prime } & = & \rho_{3}\gamma_{h}I_{A}+\sigma \alpha_{1}I_{H}+\left(\alpha_{2}+\beta \right)P_{H}-\rho_{r}R_{H}-\mu_{H}R_{H}\\ S_{R}^{\prime } & = & \Lambda_{R}-a\left(t\right)bI_{V}\frac{S_{R}}{N_{R}}-\mu_{R}S_{R}\\ I_{R}^{\prime } & = & a\left(t\right)bI_{V}\frac{S_{R}}{N_{R}}-\mu_{R}I_{R}\\ S_{V}^{\prime } & = & \Lambda_{V}-\mu_{1}a\left(t\right)cS_{V}\frac{I_{A}}{N_{H}}-a\left(t\right)cS_{V}\frac{I_{H}}{N_{H}}-a\left(t\right)cS_{V}\frac{P_{H}}{N_{H}}-\mu_{2}a\left(t\right)cS_{V}\frac{I_{R}}{N_{R}}-\mu_{V}S_{V}\\ I_{V}^{\prime } & = & \mu_{1}a\left(t\right)cS_{V}\frac{I_{A}}{N_{H}}+a\left(t\right)cS_{V}\frac{I_{H}}{N_{H}}+a\left(t\right)cS_{V}\frac{P_{H}}{N_{H}}+\mu_{2}a\left(t\right)cS_{V}\frac{I_{R}}{N_{R}}-\mu_{V}I_{V} \end{array}$$ (1)

with

$$\begin{array}{ccc} N_{H}^{\prime } & = & \Lambda_{H}-\mu_{H}N_{H}-\delta I_{H}\\ N_{R}^{\prime } & = & \Lambda_{R}-\mu_{R}N_{R}\\ N_{V}^{\prime } & = & \Lambda_{V}-\mu_{V}N_{V} \end{array}$$

### Model properties

Let *C* denote all continuous functions on the real line. Given *f* ∈ *C*, and if *f* is *ω*-periodic, then the average value of *f* on a time interval [0, *ω*] can be defined as:

$$\bar{f}=\frac{1}{\omega }\int_{0}^{\omega }f\left(t\right)dt$$

The maximum and minimum values of *f* on a time interval [0, *ω*] is denoted as *f*^*M*^ and *f*^*m*^, respectively and defined as

$$f^{M}=\max_{t\in [0,\omega ]}f\left(t\right),$$

and

$$f^{m}=\min_{t\in [0,\omega ]}f\left(t\right)$$

All parameters of the model (1) are assumed to be nonnegative. Furthermore since the above model monitors living populations, it is assumed that all the state variables are nonnegative at time *t* = 0. It is noted that in the absence of the disease (*δ* = 0), the total human population size, *N*_*H*_ → Λ_*H*_/*μ*_*h*_ as *t* → ∞, also *N*_*R*_ → Λ_*R*_/*μ*_*R*_ and *N*_*V*_ → Λ_*V*_/*μ*_*V*_ as *t* → ∞. This shows that the biologically-feasible region:

$\Omega =\{\left(S_{H},I_{A},I_{H},T_{H},P_{H},R_{H},S_{R},I_{R},S_{V},I_{V}\right)\in R_{+}^{10}:S_{H},I_{A},I_{H},T_{H},P_{H},R_{H},S_{R},I_{R},S_{V},I_{V}\geq 0,N_{H}\leq \frac{\Lambda_{H}}{\mu_{H}},N_{R}\leq \frac{\Lambda_{R}}{\mu_{R}},N_{V}\leq \frac{\Lambda_{V}}{\mu_{V}}\}$ is a positively-invariant domain, and thus, the model is mathematically well posed, and it is sufficient to consider the dynamics of the flow generated by the model in this positively-invariant domain Ω. Here $R_{+}^{10}$ denotes the non-negative cone of *R*^10^ including its lower dimensional faces. We denote the boundary and the interior of Ω by ∂Ω and $\overset{\circ }{\Omega },$ respectively.

Let $m=\frac{N_{V}}{N_{H}}$ be the female sandfly vector human ratio defined as the number of female sandflies per human host. Here, *m* is taken as a constant because it is well known that a vector takes a fixed number of blood meals per unit time independent of the population density of the host. Similarly, we let $n=\frac{N_{V}}{N_{R}}$ be the female sandfly vector reservoir ratio defined as the number of female sandflies per reservoir host.

Disease free equilibrium calculation and its stability analysis, mathematical description of basic reproduction number (*R*_0_), existence and permanence of endemic periodic solution and its global stability has been discussed as separate sections in Section A in S1 File.

### Model calibration (Case study)

To estimate model parameters, we use the monthly VL infected case records reported in South Sudan for the year 2012 [36]. The estimated parameters from the data are -

- *δ*_*r*_ (The amplitude of seasonality)
- b (Transmission probability of VL in human and reservoir population)
- *k*_1_ (Total number of reservoir per human)
- *k*_2_ (Total number of sandfly per human)
- c (Transmission probability of VL in sandfly population)

The initial human demographic parameters *S*_*H*_(0), *I*_*H*_(0), *P*_*H*_(0), *T*_*H*_(0), *R*_*H*_(0) as well as initial infected reservoir population *I*_*R*_(0), initial infected sandfly population *I*_*V*_(0) were also estimated.

We assume that *I*_*A*_(0) = *N*_*H*_(0) − *S*_*H*_(0) − *I*_*H*_(0) − *T*_*H*_(0) −*P*_*H*_(0) − *R*_*H*_(0) and *T*_*H*_(0) = *C*(0) − *I*_*H*_(0) − *P*_*H*_(0).

The carrying capacity (*N*_*V*_) of the sandfly population is taken to be a multiple of the total human population at the beginning, i.e. *N*_*V*_(0) = *k*_2_ × *N*_*H*_(0), where *k*_2_ is the total number of sandfly per human. Similarly, initially *N*_*R*_(0) = *k*_1_ × *N*_*H*_(0), where *k*_1_ is the total number of reservoir per human. We estimated *k*_1_ and *k*_2_ from the given data of Visceral Leishmaniasis. Initial susceptible sandfly population *S*_*V*_(0) = *N*_*V*_(0) − *I*_*V*_(0) and initial susceptible reservoir population *S*_*R*_(0) = *N*_*R*_(0) − *I*_*R*_(0).

According to the [36], 28,328 new cases of VL were reported from September 2009 until December 2012. Relapses represented 8.6% cases in 2012 and PKDL was noted in 4.6% of patients in 2012. We added a compartment *I*_*C*_ to our model (1) to calculate the cumulative number of new notified VL infections. The number of new VL cases *I*_*C*_ (symptomatic infected *I*_*H*_+PKDL infected *P*_*H*_+Transient *T*_*H*_) from the model (1) has the following form

$$\frac{dI_{C}}{dt}=\rho_{1}\gamma_{h}I_{A}+\left(1-\sigma \right)\alpha_{1}I_{H}+\delta_{p}T_{H}+\rho_{2}\gamma_{h}I_{A},$$

which represents the rate of cumulative new VL cases. Here, *ρ*_1_ γ_*h*_ *I*_*A*_ represents new symptomatic infected *I*_*H*_, (1 − *σ*)*α*_1_ *I*_*H*_ represents new Transient *T*_*H*_ and *δ*_*p*_ *T*_*H*_ + *ρ*_2_ γ_*h*_ *I*_*A*_ represents new PKDL infected *P*_*H*_ population.

While simulating our model (1) with above mentioned assumptions, the numerical solutions for the above equation gives the predicted monthly cumulative VL incidence. For performing numerical simulations, ode15s and ode45 MATLAB ODE solvers were used. Here, *I*_*C*_(0) = number of new notified cases at the first time point of the data (C(0)).

We minimize the sum of the squared error between the model and data, which is given by

$$RSS\left(\hat{\theta }\right)=\Sigma_{i=1}^{n}{\left(Y_{i}-I_{C}\left(t_{i},\hat{\theta }\right)\right)}^{2},$$

where *Y*_*i*_ is the cumulative VL data, and $Y_{i}=I_{C}\left(t_{i},\hat{\theta }\right)+\epsilon$, *ϵ* ∼ *N*(0, *Iσ*^2^) where *t*_*i*_ = 0, 31, 60, 91… days [39], and *ϵ* be the error of fit, which follows an independent Gaussian distribution having unknown variance *σ*^2^.

The prior distribution can be viewed as representing the current state of knowledge, or uncertainty, about the model parameters prior to data being observed. From previous literature, we observe that all our unknown parameters are non negative and bounded. It is more realistic to assume that the prior distributions are “bell curve” shape rather than flat one. So, an independent Gaussian prior specification is assumed for the unknown parameters $\hat{\theta }$ [41] of the model (1) i.e. *θ*_*j*_ ∼ *N*(*ν*_*j*_, *ψ*_*j*_), where j = 1,2,3,……N. Realizations of Gaussian processes with a proper covariance function can provide nearly all functions we can encounter in “real life”. Also, they are convenient and provide exact inference and marginal distribution.

We also assume that the inverse of the error variance follows a gamma distribution as prior with the following form:

$$\rho \left(\sigma^{-2}\right)∼\Gamma \left(\frac{n_{0}}{2},\frac{n_{0}S_{0}^{2}}{2}\right),$$

where $S_{0}^{2}$, *n*_0_ are the prior mean and prior accuracy of *σ*^2^, respectively.

Using conditional conjugacy property of Gamma distribution, the conditional distribution is $\rho (\sigma^{-2}|Y,\hat{\theta })$ also a Gamma distribution with

$$\rho \left(\sigma^{-2}|Y,\hat{\theta }\right)=\Gamma \left(\frac{n_{0}+n}{2},\frac{n_{0}S_{0}^{2}+RSS\left(\hat{\theta }\right)}{2}\right).$$

This conditional conjugacy property makes it possible to sample and update within each Metropolis-Hastings simulation step for the other parameters. Since, we assume independent Gaussian prior specification for $\hat{\theta }$, therefore we can now calculate the prior sum-of-squares for the given $\hat{\theta }$ as:

$$RSS_{pri}\left(\hat{\theta }\right)=\Sigma_{i=1}^{R}{\left[\frac{\theta_{i}-\nu_{i}}{\psi_{i}}\right]}^{2}$$

Then, for a fixed value of *σ*^2^, the posterior distribution of $\hat{\theta }$ is as follows:

$$\rho \left(\hat{\theta }|Y,\sigma^{2}\right)\propto exp\left[-\frac{1}{2}\left(\frac{RSS(\hat{\theta })}{\sigma^{2}}+RSS_{pri}(\hat{\theta }\right)\right)]$$ (2)

and the posterior ratio needed in the Metropolis-Hastings acceptance probability can be written as:

$$\frac{\rho (\hat{\theta^{1}}|Y,\sigma^{2})}{\rho (\hat{\theta^{2}}|Y,\sigma^{2})}=exp\left[-\frac{1}{2}\left(\left(\frac{RSS(\hat{\theta^{1}})}{\sigma^{2}}-\frac{RSS(\hat{\theta^{2}})}{\sigma^{2}}\right)+\frac{1}{2}\left(RSS_{pri}(\hat{\theta^{2}}\right)+RSS_{pri}(\hat{\theta^{1}}\right)\right))].$$ (3)

MCMC tool box in MATLAB version R2011b was used to estimate the unknown $\hat{\theta }$ for the model [41, 42]. Geweke’s Z-scores [43] were used to ensure the chain convergence (Table D in S1 File).

The estimated model parameters, including human, reservoir and sandfly demographic parameters, for South Sudan in 2012 are given in Table A and B of S1 File. Further, plots for the posterior distributions of the estimated parameters, including human, reservoir and sandfly demographic parameters of the model (1) are given in S1.A Fig. The basic reproductive number (*R*_0_), the expected number of secondary cases produced by a single infection in a completely susceptible population, was also calculated for each parameter set so as to get a posterior distribution of *R*_0_ (S1.B Fig). Also, trace plots of parameters (S1.C Fig) were generated to identify whether the Markov chain has converged or not.

### Model sensitivity analysis

Using the standard combination of Latin Hypercube Sampling (LHS) and Partial Rank Correlation Coefficient (PRCC) multivariate analysis, we performed the sensitivity analysis for the model (1). LHS is a stratified Monte Carlo sampling method, where the random parameter distributions are divided into N equal probability intervals and samples are taken from each [44], where N is the sample size. PRCC is an efficient method for measuring the nonlinear, monotonic relationship between inputs and the model outcome of interest.

The inputs are the estimated parameters as given in the “Model calibration” section and as described “Parameter Estimation” of Section C in S1 File. The sensitivity analysis was performed for 2000 random samples of each parameter in the model for ranges given in Table A of S1 File. The details of sensitivity analysis are discussed in the “Sensitivity Analysis—Results” section of Section C in S1 File. Whereas, the model outcome is the cumulative proportion of infectious individuals, which is the solution of $\frac{dI_{C}}{dt}=\rho_{1}\gamma_{h}I_{A}+\left(1-\sigma \right)\alpha_{1}I_{H}+\delta_{p}T_{H}+\rho_{2}\gamma_{h}I_{A}$ and *R*_0_.

### The optimal control problem

#### Construction of the problem

We incorporated three different popularly used VL intervention strategies in the model (1) namely, the use of treated bed nets, treatment of infective individuals using antibiotics and spray of insecticides [6]. The control functions, *u*_1_, *u*_2_, *u*_3_ and *u*_4_; represent time dependent efforts of use of treated bednets, treatment of symptomatic KA patients, treatment of PKDL patients and spray of insecticides respectively. The controls are practiced on a time interval [0; *t*_*f*_], where *t*_*f*_ is the final time.

System of non-linear differential equations representing the effect of different interventions on our basic model (1) is given as follows:

$$\begin{array}{ccc} S_{H}^{\prime } & = & \Lambda_{H}-a\left(t\right)bI_{V}\frac{S_{H}}{N_{H}}\left(1-u_{1}\left(t\right)\right)-\mu_{H}S_{H}+\rho_{r}R_{H}\\ I_{A}^{\prime } & = & a\left(t\right)bI_{V}\frac{S_{H}}{N_{H}}\left(1-u_{1}\left(t\right)\right)-\left(\gamma_{h}+\mu_{H}\right)I_{A}\\ I_{H}^{\prime } & = & \rho_{1}\gamma_{h}I_{A}-\left(u_{2}\left(t\right)+\delta +\mu_{H}\right)I_{H}\\ T_{H}^{\prime } & = & \left(1-\sigma \right)u_{2}\left(t\right)I_{H}-\left(\delta_{p}+\mu_{H}\right)T_{H}\\ P_{H}^{\prime } & = & \rho_{2}\gamma_{h}I_{A}+\delta_{p}T_{H}-\left(u_{3}\left(t\right)+\beta +\mu_{H}\right)P_{H}\\ R_{H}^{\prime } & = & \rho_{3}\gamma_{h}I_{A}+\sigma u_{2}\left(t\right)I_{H}+\left(u_{3}\left(t\right)+\beta \right)P_{H}-\rho_{r}R_{H}-\mu_{H}R_{H}\\ S_{R}^{\prime } & = & \Lambda_{R}-a\left(t\right)bI_{V}\frac{S_{R}}{N_{R}}-\mu_{R}S_{R}\\ I_{R}^{\prime } & = & a\left(t\right)bI_{V}\frac{S_{R}}{N_{R}}-\mu_{R}I_{R}\\ S_{V}^{\prime } & = & \Lambda_{V}-\left(\mu_{1}a\left(t\right)cS_{V}\frac{I_{A}}{N_{H}}+a\left(t\right)cS_{V}\frac{I_{H}}{N_{H}}+a\left(t\right)cS_{V}\frac{P_{H}}{N_{H}}\right)\left(1-u_{1}\left(t\right)\right)\\ & - & \mu_{2}a\left(t\right)cS_{V}\frac{I_{R}}{N_{R}}-\left(\mu_{V}+u_{4}\left(t\right)\right)S_{V}\\ I_{V}^{\prime } & = & \left(\mu_{1}a\left(t\right)cS_{V}\frac{I_{A}}{N_{H}}+a\left(t\right)cS_{V}\frac{I_{H}}{N_{H}}+a\left(t\right)cS_{V}\frac{P_{H}}{N_{H}}\right)\left(1-u_{1}\left(t\right)\right)\\ & + & \mu_{2}a\left(t\right)cS_{V}\frac{I_{R}}{N_{R}}-\left(\mu_{V}+u_{4}\left(t\right)\right)I_{V} \end{array}$$ (4)

where the initial conditions *S*_*H*_(0), *I*_*A*_(0), *I*_*H*_(0), *T*_*H*_(0), *P*_*H*_(0), *R*_*H*_(0), *V*(0), *S*_*R*_(0), *I*_*R*_(0), *S*_*V*_(0), *I*_*V*_(0) and the above model parameters are listed in Table A and Table B of S1 File. The control functions *u*_1_(*t*), *u*_2_(*t*), *u*_3_(*t*) and *u*_4_(*t*) are bounded and Lebesgue integrable functions.

Our control problem involves that in which the number of infected individuals with visceral leishmaniasis and the cost of applying different controls are minimized subject to the system (5). The objective function to be minimized is defined as

$$J\left(u_{1},u_{2}\right)=\int_{0}^{t_{f}}\left(A_{1}I_{A}\left(t\right)+AI_{H}\left(t\right)+A_{2}P_{H}\left(t\right)+\frac{1}{2}Bu_{1}^{2}+\frac{1}{2}Cu_{2}^{2}+\frac{1}{2}Du_{3}^{2}+\frac{1}{2}Eu_{4}^{2}\right)e^{-\sigma_{1}t}dt;$$ (5)

subject to the state Eq (5) and the total cost is given by

$$C_{VL}=\int_{0}^{t}\left(c_{b}u_{1}S_{H}+c_{t1}u_{2}I_{H}+c_{t2}u_{3}P_{H}+c_{v}u_{4}\left(S_{V}+I_{V}\right)\right)\;dt,;$$ (6)

where *t*_*f*_ is the final time, *σ*_1_ is the discount rate applied to future years and *A*_1_, *A* and *A*_2_ are weight constants of the *I*_*A*_, *I*_*H*_, *P*_*H*_ group, respectively, whereas, B, C, D and E are weight constants for treated bed nets, treatment (for *I*_*H*_, *P*_*H*_) and spray of insecticide efforts respectively which regularize the optimal control. The discounting procedure reflects inherent uncertainty about the future. It is assumed that, there is no linear relationship between the coverage of these interventions and their corresponding costs [45]. We seek an optimal control $u_{1}^{*}\left(t\right)$, $u_{2}^{*}\left(t\right)$, $u_{3}^{*}\left(t\right)$ and $u_{4}^{*}\left(t\right)$ such that

$$J\left(u_{1}^{*},u_{2}^{*},u_{3}^{*},u_{4}^{*}\right)=\min\left\{J\left(u_{1},u_{2},u_{3},u_{4}\right)|u_{1},u_{2},u_{3},u_{4}\in U\right\}$$ (7)

where *U* = {(*u*_1_(*t*), *u*_2_(*t*), *u*_3_(*t*), *u*_4_(*t*))|(*u*_1_(*t*), *u*_2_(*t*), *u*_3_(*t*), *u*_4_(*t*)) measurable, *a*_*i*_ ≤ *u*_*i*_(*t*)≤*b*_*i*_, *i* = 1, 2, 3, 4, *t* ∈ [0, *t*_*f*_]} is the control set.

#### Analysis of the optimal control problem

The necessary conditions that an optimal control must satisfy, come from the Pontryagin’s Maximum Principle [46]. The Hamiltonian H, with respect to *u*_1_, *u*_2_, *u*_3_ and *u*_4_ can be written as:

$$\begin{array}{ccc} H & = & \left(A_{1}I_{A}\left(t\right)+AI_{H}\left(t\right)+A_{2}P_{H}\left(t\right)+\frac{1}{2}Bu_{1}^{2}+\frac{1}{2}Cu_{2}^{2}+\frac{1}{2}Du_{3}^{2}+\frac{1}{2}Eu_{4}^{2}\right)\\ & + & \sum_{i=1}^{10}\lambda_{i}g_{i}+\lambda_{C_{VL}}\left\{c_{b}u_{1}S_{H}+c_{t1}u_{2}I_{H}+c_{t2}u_{3}P_{H}+c_{v}u_{4}\left(S_{V}+I_{V}\right)\right\} \end{array}$$ (8)

where *g*_*i*_ is the right hand side of the differential equation of the *i*^*th*^ state variable and λ_*i*_ are the adjoint variables. By applying Pontryagin’s Maximum Principle [46, 47] we get the following result:

**Proposition 1** *There exists an optimal control* $u_{1}^{*}$, $u_{2}^{*}$, $u_{3}^{*}$ *and* $u_{4}^{*}$ *and corresponding solution*, $S_{H}^{*}$, $I_{A}^{*}$, $I_{H}^{*}$, $T_{H}^{*}$, $P_{H}^{*}$, $R_{H}^{*}$, $S_{R}^{*}$, $I_{R}^{*}$, $S_{V}^{*}$ *and* $I_{V}^{*}$, *that minimizes J*(*u*_1_, *u*_2_, *u*_3_, *u*_4_) *over U. Furthermore, there exists adjoint functions*, $\lambda_{1}(t),\;\ldots ,\;\lambda_{10}(t),\;\lambda_{C_{V\;L}}(t)$ *such that*

$$\frac{\partial \lambda_{i}}{\partial t}=f_{i},i=1,.....,10\;and\;\frac{\partial \lambda_{C_{VL}}}{\partial t}=0.$$ (9)

*with transversality conditions*

$$\lambda_{i}\left(t_{f}\right)=0,i=1,.....,10\;and\;\lambda_{C_{VL}}\left(t_{f}\right)=0.$$ (10)

*and*

$$\begin{array}{ccc} u_{1}^{*} & = & \min\left\{b_{1},\max\left[a_{1},c_{1}\right]\right\} \end{array}$$ (11)

*and*

$$\begin{array}{ccc} u_{2}^{*} & = & \min\left\{b_{2},\max\left[a_{2},c_{2}\right]\right\} \end{array}$$ (12)

*and*

$$\begin{array}{ccc} u_{3}^{*} & = & \min\left\{b_{3},\max\left[a_{3},c_{3}\right]\right\} \end{array}$$ (13)

*and*

$$\begin{array}{ccc} u_{4}^{*} & = & \min\left\{b_{4},\max\left[a_{4},c_{4}\right]\right\} \end{array}$$ (14)

*where*

$$\begin{array}{ccc} c_{1} & = & \frac{\left(\lambda_{2}-\lambda_{1}\right)a\left(t\right)b\frac{I_{V}S_{H}}{N_{H}}+(\mu_{1}a\left(t\right)c\frac{I_{A}S_{V}}{N_{H}}+a\left(t\right)c\frac{P_{H}S_{V}}{N_{H}}+{c_{1}}^{{}^{\prime }}}{2Be^{-\sigma_{1}t}}\\ {c_{1}}^{{}^{\prime }} & = & a\left(t\right)c\frac{I_{H}S_{V}}{N_{H}})\left(\lambda_{9}-\lambda_{10}\right)-\lambda_{C_{VL}}c_{b}S_{H}\\ c_{2} & = & \frac{\left(\lambda_{3}-\left(1-\sigma \right)\lambda_{4}-\sigma \lambda_{6}\right)I_{H}-c_{t1}I_{H}\lambda_{C_{VL}}}{2Ce^{-\sigma_{1}t}}\\ c_{3} & = & \frac{\left(\lambda_{6}-\lambda_{5}\right)P_{H}-c_{t2}P_{H}\lambda_{C_{VL}}}{2De^{-\sigma_{1}t}}\\ c_{4} & = & \frac{\left(\lambda_{9}S_{V}+\lambda_{10}I_{V}\right)-c_{v}\left(I_{V}+S_{V}\right)\lambda_{C_{VL}}}{2Ee^{-\sigma_{1}t}} \end{array}$$

Details of adjoint functions $\frac{\partial \lambda_{i}}{\partial t}$ is given in the “Optimal Control” Section A of S1 File.

#### Numerical simulations

The optimality system is a two-point boundary problem, because of the initial condition on the state system (5), and the terminal condition on the adjoint system (10). Using the values for the parameters given in Tables A and B in S1 File, and the initial conditions in Table C in S1 File, we first solve the initial valued state system (5) forward in time, using an initial guess for the above defined control functions (11–14). Then, using the same initial guess for the control functions, we solve the adjoint system (10) with terminal conditions backward in time. The controls are updated at each iteration using the optimality conditions (11–14) The iterations continue until the system converges.

### Cost-effectiveness analysis

Although the choice of optimal combinations of control strategies and need for a proper implementation is important, it is also equally important to choose a combination that is also cost-effective while implementing on a large scale. Cost-effectiveness analysis is a method to evaluate the benefits associated with interventions in infections (treatment of infected individuals, use of treated bednets and spray of insecticides)with respect to the strategy’s involved cost [45]. To calculate the cost-effectiveness of our strategies, we used two methods, namely the infection averted ratio (IAR) and the incremental cost-effectiveness ratio (ICER).

#### IAR

The infection averted ratio (IAR) can be defined as the ratio of number of infections averted to the number of recovered. The number of infection averted is given as the difference between the total number of infective individuals present without control and total infective individuals present with control.

#### ICER

The incremental cost-effectiveness ratio (ICER) can be defined as the additional cost per additional health outcome. ICER is an incremental ratio of the difference in total cost between one strategy and the next best alternative to the difference in total number of averted infections through each strategy.

## Results and discussion

### Model fitting and validation

**Fig 1:**
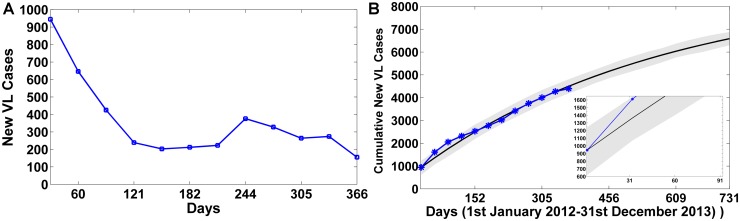
Model fitting and validation. A) Number of VL cases reported for South Sudan for the period January 2012–December 2012, B) Observed cumulative data and the output of the fitted model—Cumulative new VL cases (blue star) from the data, and model simulated data (thick black curve) are plotted using estimated parameter values from Tables A, B and initial conditions from Table C in S1 File, for the time period (1st January 2012–31st December 2012). The model was further simulated upto December 2013, the next year for signifying the predictive power of the model.

Monthly VL infected cases reported in South Sudan for the year 2012 [36] was chosen to estimate our model parameters. The data shows a peak during months of Jan-Feb, after which the reported VL cases decline to lower numbers (Fig 1A). This data was chosen such that the model predictions using the estimated model parameters almost accurately fit with the cumulative number of VL cases reported for the year 2012. The cumulative number of cases are almost accurately predicted by the model for the year 2012 (Fig 1B). Further, using the fitted model, predictive simulations for the next year Jan 2013–Dec 2013 was also performed to signify the use of the model to predict future VL cases. Our model predicted that cumulatively 4,394 and 6,587 persons are diagnosed as new KA cases at the end of December 2012 and December 2013 respectively. Thus, approximately 6,587−4,394 = 2,193 new KA cases were predicted for the year 2013. WHO [48] reported that around 2,364 new VL cases were reported in South Sudan for the year 2013. The initial values for the simulations were taken according to the reported yearly census data of South Sudan. The total human populations (*N*_*H*_) obtained after simulations for the 2012 and 2013 cases are indicated in S6 Fig and is suggestive of around 4.3% yearly growth of population in South Sudan, which is also comparable with the reported population growth (approximately 4% yearly). The cumulative number of predicted VL cases from our model were comparable to the actual reported VL cases in South Sudan for 2013 further substantiating the predictive power of the model.

Note that the above mentioned simulations were performed assuming a periodic sandfly biting rate due to the periodic nature of the data, which was further verified using seasonality test. Performing the seasonality test, the null hypothesis that “there is no effect of seasonality on disease dynamics” was rejected at a significance level of p<0.05, thus, supporting the notion of seasonality influence on VL disease dynamics, modeled as a periodic sandfly biting rate, albeit with low amplitude of seasonality (see Section B in S1 File for details).

### Choice of optimal control strategy combinations

The most popularly used VL intervention strategies include the treatment of infected, use of treated bed nets and spray of insecticides for vector control [6]. Initially, each control strategy and its effect on VL disease transmission was investigated individually. S3 S4 and S5 Figs display the model-based predictions of effects of different intervention strategies on a yearly basis and its comparison with a scenario where no optimal control is introduced in the population. Simulations were performed on the previously standardized model with estimated parameters (Tables A and B in S1 File) and initial values (Table C in S1 File) to compare the normal and optimally treated scenarios. Model predictions suggest that the treatment policy is very useful to optimally control KA infected individuals (S3.D Fig). As the number of humans harboring infection reduce due to medical treatment, susceptible sandfly vectors are also deprived of infective human hosts for their blood meal and hence, do not become infected. The amount of infected vector populations thus, reduce exponentially, although the rate at which they decrease is low (S3.E Fig). The optimal use of treated bednets on the other hand performs even worse as sandfy populations reduce only intermittently, after which the rate of infected vectors increase(S4.E Fig). Similarly, optimal use of insecticides are relatively weaker in controlling infected populations as compared to both the aforementioned policies (S5 Fig).

It is important to note here that none of these policies are by themselves effective enough in reducing VL infection spread and hence, require better strategies to combat the VL infection. We propose from our model that instead of using each of these strategies separately, optimal combinations of the aforementioned control strategies would be better alternatives to reduce VL disease spread. So as to test this contention, we introduced different optimal combination strategies in our model and numerically compare their effects on infected populations.

We devise and test for the following combinations:

1. Strategy A: combination of use of treated bednets, treatment of infective individuals and spray of insecticides.
2. Strategy B: combination of use of treated bednets and treatment of infective individuals.
3. Strategy C: combination of use of treated bednets and spray of insecticides.
4. Strategy D: combination of treatment of infective individuals and spray of insecticides.

#### Strategy A

**Fig 2:**
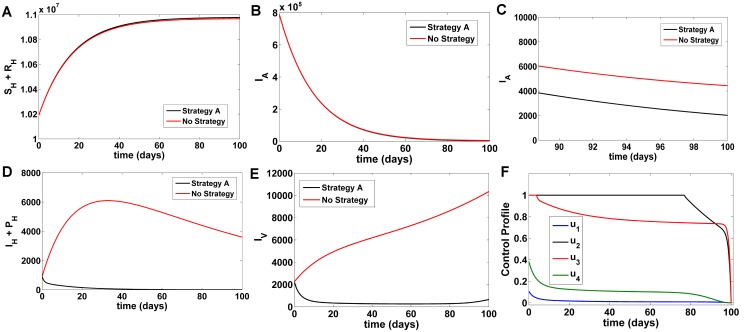
Comparison of Strategy A with a scenario without any control. A) Number of susceptible and recovered humans B) Number of asymptomatic KA infected humans C) Fig 2B (magnified, t = 90–100 days) D) Number of symptomatic KA and PKDL humans E) Number of infected vector populations F) Numerical solutions for optimal control functions used in Strategy A.

Under this strategy, we use all the four controls *u*_1_, *u*_2_, *u*_3_ and *u*_4_ to optimize the objective function *J*. Comparing Strategy A with a situation where no control strategy was used, it was observed that the susceptible and recovered human populations (*S*_*H*_ + *R*_*H*_) increase in number (Fig 2A), asymptomatic KA populations (*I*_*A*_) marginally reduce (Fig 2B and 2C), symptomatic KA and PKDL (*I*_*H*_ + *P*_*H*_) reduce at an exponential rate (Fig 2D), and infected sandfly populations (*I*_*V*_) (Fig 2E) decreases significantly at a near exponential rate where sandfly populations reduce below 1000 within 15 days. At *t* = 100 days, comparing Strategy A with no strategy, there is an increase in *S*_*H*_ + *R*_*H*_ by 9975 individuals, decrease in *I*_*A*_ by 2409 individuals and *I*_*V*_ by 9694 individuals (Fig 2) respectively. With this strategy, *I*_*H*_ + *PKDL* will be eliminated from the system within *t* = 50 days in humans. The control profile shown in Fig 2F, shows that the control *u*_1_ is at 10% initially, after that it drops slowly to the lower bound while control *u*_2_ and *u*_3_ decreases from the maximum of 100% to the lower bound in 98 days and the control *u*_4_ is at 36% for beginning time before dropping slowly to the lower bound in the 92^*th*^ day. This suggests that a low effort is required on treated bednet and insecticide spray under this strategy.

#### Strategy B

**Fig 3:**
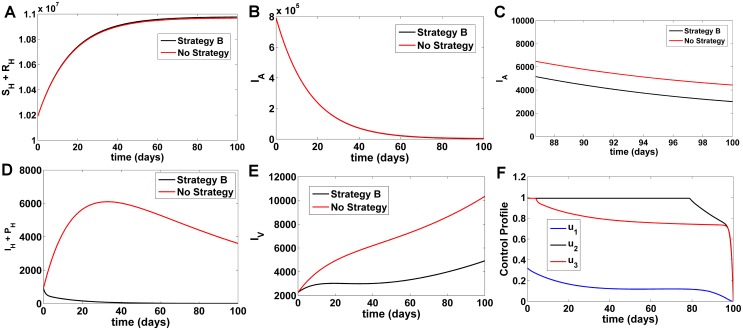
Comparison of Strategy B with a scenario without any control. A) Number of susceptible and recovered humans B) Number of asymptomatic KA infected humans C) Fig 3B (magnified, t = 90–100 days) D) Number of symptomatic KA and PKDL humans E) Number of infected vector populations F) Numerical solutions for optimal control functions used in Strategy B.

In this strategy, the treated bednet control *u*_1_ and the treatment control *u*_2_, *u*_3_ are used to optimize the objective function *J* while we set the spray of insecticides control *u*_4_, to zero. Comparing Strategy B with a situation where no control strategy was used, it was observed that the susceptible and recovered population (*S*_*H*_ + *R*_*H*_) increase in number (Fig 3A), asymptomatic KA populations (*I*_*A*_) marginally reduce (Fig 3B and 3C) suggesting that asymptomatics become symptomatic after which they are cured, and symptomatic KA, PKDL (*I*_*H*_ + *P*_*H*_) cases reduce at an exponential rate (Fig 3D) very similar to Strategy A. Infected sandfly populations (*I*_*V*_) (Fig 3E) decreases significantly after introduction of Strategy B, albeit at a rate slower than Strategy A (Fig 3E). At *t* = 100 days, comparing Strategy B with no strategy, there is an increase in the *S*_*H*_ + *R*_*H*_ by 8991 individuals and decrease in *I*_*A*_ by 1429 individuals, *I*_*H*_ + *P*_*H*_ by 3596 individuals, and *I*_*V*_ by 5430 individuals respectively (Fig 3). In Fig 3F, the control *u*_2_, *u*_3_ is at the upper bound of 100% and drops gradually until reaching the lower bound, while control on treated bednets *u*_1_ is at the maximum of 32% initially before dropping gradually to the lower bound in the 98th day. This suggests that there is a low effort for the use of treated bednets and a higher effort required for medical treatment of individuals under this strategy.

#### Strategy C

**Fig 4:**
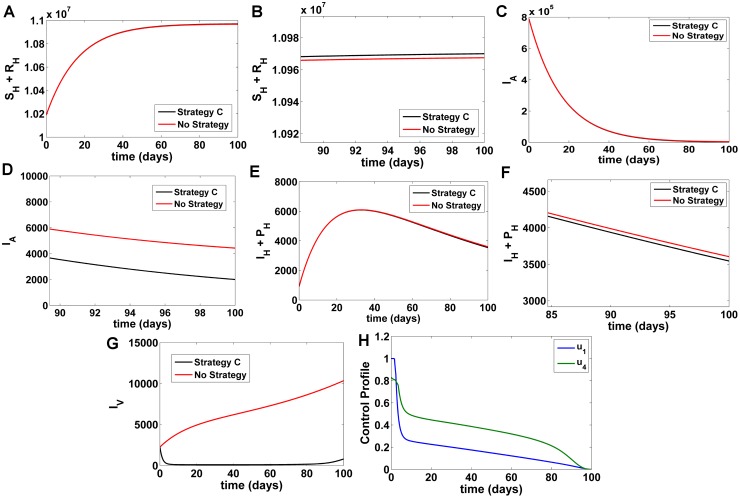
Comparison of Strategy C with a scenario without any control. A) Number of susceptible and recovered humans B) Fig 4A (magnified) C) Number of asymptomatic KA infected humans D) Fig 4C (magnified, t = 90–100 days) E) Number of symptomatic KA and PKDL humans F)Fig 4E (magnified, t = 90–100 days) G) Number of infected vector populations H) Numerical solutions for optimal control functions used in Strategy C.

Here under this strategy treated bednet control *u*_1_ and the spray of insecticide control *u*_4_ are used to optimize the objective function *J* while we set treatment control *u*_2_, *u*_3_ = 0. Comparing Strategy C with a situation where no control strategy was used, it was observed that the susceptible and recovered population (*S*_*H*_ + *R*_*H*_)(Fig 4A and 4B) increase and the numbers of infected humans *I*_*A*_ (Fig 4C and 4D), *I*_*H*_ + *PKDL* (Fig 4E and 4F) with optimal strategy show only a marginal decrease as compared to the numbers in the case without control. The number of infected humans are reduced by a low amount in this strategy when compared with Strategy A and B. Infected sandflies *I*_*V*_ (Fig 4G) reduce to considerably low values which is better than any other combination strategy. At *t* = 100 days, comparing Strategy C with no strategy, *S*_*H*_ + *R*_*H*_ increase by 2511, *I*_*A*_ decrease by 2426, *I*_*H*_ + *P*_*H*_ decrease by 59 and *I*_*V*_ decrease by 9553 individuals respectively (Fig 4). The control profile is shown in Fig 4H; we see that both the control *u*_1_ and *u*_4_ start from their upper bounds steadily decline and converge to its lower bound within 96 days. This suggests that there is a low effort involved for the use of both treated bednets and spray of insecticides when applied in combination.

#### Strategy D

**Fig 5:**
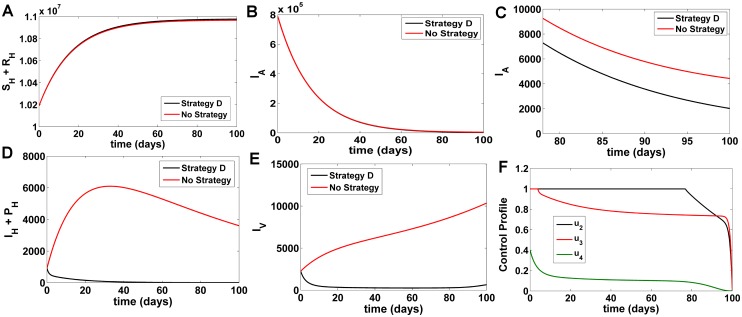
Comparison of Strategy D with the no control scenario. A) Number of susceptible and recovered humans B) Number of asymptomatic KA infected humans C) Fig 5B (magnified, t = 90–100 days) D) Number of symptomatic KA and PKDL humans E) Number of infected vector populations F) Numerical solutions for optimal control functions used in Strategy D.

Under this strategy, we optimize the objective function *J* using the treatment control *u*_2_, *u*_3_ and the spray of insecticides controls *u*_4_ while the treated bed net control *u*_1_ = 0. Comparing Strategy D with the no control situation, it was observed that the susceptible and recovered population (*S*_*H*_ + *R*_*H*_) significantly increased Fig 5A, the asymptomatic population *I*_*A*_ (Fig 5B and 5C) also decreased, *I*_*H*_ + *PKDL* (Fig 5D) and infected sandflies *I*_*V*_ (Fig 5E) also reduce tremendously. Strategy D decreases the infected human and infected sandfly population drastically while increase the number of susceptible and recovered human population (Fig 5). At *t* = 100 days, comparing Strategy D with no strategy, *S*_*H*_ + *R*_*H*_ increase by 9974 individuals, *I*_*A*_ decrease by 2405 individuals and *I*_*V*_ decrease by 9696 individuals respectively. With this strategy, *I*_*H*_ + *PKDL* will be eliminated from the system within t = 50 days in humans. Fig 5F highlights the optimal control profile for strategy D. It was observed that the treatment control *u*_2_, *u*_3_ starting from its upper bound drop at a very slow rate and reach its lower bound taking around 96 days while *u*_4_ is at the maximum of 40% before dropping to the lower bound in the 95 days. This again suggests that the spray of insecticide controls require less effort for implementation when used in combination with treatment policies.

### Effect on *R*_0_

**Fig 6:**
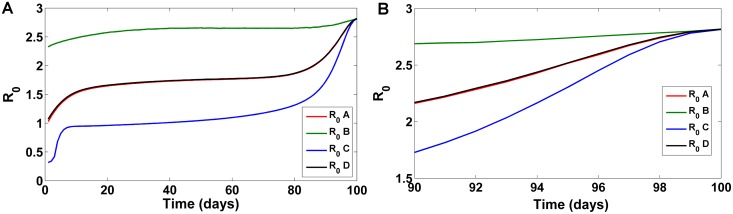
Effect of control strategies on *R*_0_. A) Comparison of *R*_0_ behavior for the four strategies B) Fig 6A magnified for t = 90–100 days.

The above applied optimal control strategies applied in our model are unable to predict the long term dynamics of the disease as a whole. As and when the intervention strategies are stopped, a few remaining infectious people/vectors can initiate a new outbreak of the disease [28]. In the context of epidemiology, the basic reproduction number (*R*_0_), that describes the number of secondary infections arising from a single individual during period of infection, is an effective measure for understanding long term endemicity [49]. Hence, the effect of applied strategies on *R*_0_ was investigated. Fig 6 describes the numerical simulation results of *R*_0_ under different control strategy combinations. Assuming that optimal control combinations are implemented in the beginning of the year; in the early stages, strategy C performs well and suppresses the spread of disease, strategy B performs almost similarly throughout and do not affect *R*_0_ indicating long term persistence of disease for these two combinations, followed by strategies A and D. Eventually after completing 100 days, as the provided optimal control has reached its lower bound, *R*_0_ increases displaying disease persistence (Fig 6B). This analysis provides us with the fact that implementation of intervention strategies at different time points leads to different disease dynamics. Hence, it is important to determine the time point at which optimal control needs to be implemented. Also, it further points to the fact that complete elimination of the disease by any control strategy combination can be achieved only if the control strategies continue for long periods of time (determined by the upper bounds of the controls *u*_1_, *u*_2_, *u*_3_ and *u*_4_).

### Cost-effectiveness of the suggested strategies

In order to understand the cost-effectiveness of each of the optimal combinations, ICER and IAR were calculated for each strategy (see “Cost-effective analysis” in Methods section). The cost involved for the treatment strategies on a per person basis are given in Table B of S1 File. The comparisons of the calculated ICER and IAR for the different scenarios are given in Table 1. The lowest ICER and highest IAR for strategy D indicates that strategy D outperforms all other intervention strategies with respect to cost-effectiveness and efficiency. Strategy B is the next best strategy as it has the second lowest ICER and second highest IAR. Strategy A is also effective in eliminating the disease but has highest involved cost for implementation (highest ICER), and hence, is the least cost-effective strategy. Strategy C performs worst with respect to effective disease control (lowest IAR) and is also ineffective with respect to the involved costs (second highest ICER).

**Table 1:** ICER and IAR calculation: Strategies ranked in order of increased effectiveness.

Strategies	Total infection averted	Cost	ICER	IAR
No strategy	0	0	-	-
C	2485	7.09 × 10^5^	285.31	0.0059
B	3812	8.60 × 10^5^	112.79	0.0088
D	4794	3.35 × 10^3^	−872.35	0.0111
A	4795	7.24 × 10^4^	6.91 × 10^4^	0.0111

where the ICER is calculated as follows:

$$\begin{array}{ccc} ICER(C) & = & \frac{7.09\times 10^{5}}{2485}=285.31\\ ICER(B) & = & \frac{8.60\times 10^{5}-7.09\times 10^{5}}{3812-2485}=112.79\\ ICER(D) & = & \frac{3.35\times 10^{3}-8.60\times 10^{5}}{4794-3812}=-872.35\\ ICER(A) & = & \frac{7.24\times 10^{4}-3.35\times 10^{3}}{4795-4794}=6.91\times 10^{4} \end{array}$$

## Conclusion

Visceral leishmaniasis, being a deadly tropical disease requires community efforts and large-scale elimination programmes for effective control of disease spread among populations. Although efforts are being taken, the choice of the best strategy or combination of strategies, that largely depends on the regional, seasonal and temperature variations still remains a formidable task at hand [6]. In this paper, we introduce a general non-autonomous anthroponotic visceral leishmaniasis model that considers the human (infected compartments divided into symptomatic, asymptomatic, PKDL-infected classes) and sandfly populations and probe further to understand the effect of optimal control strategy combinations on the infected populations considered within the model. To capture the realistic situations, we considered the effect of seasonal variations on the biting rate of sandfly as an integrable periodic function in the model. Further, we mathematically analyse the model to derive the basic reproduction number *R*_0_ for the model and show that the disease-free equilibrium of the proposed model is globally asymptotically stable if *R*_0_ < 1. If *R*_0_ > 1, then the proposed system has at least one positive periodic solution, and the solution is uniformly persistent. We have also proven that if *R*_0_ > 1, then the positive periodic solution is globally asymptotically stable. We also numerically analyze the model by estimating parameters fitted for the VL cases reported in South Sudan for the year 2012 [36]. The estimated value of basic reproduction number (*R*_0_) in periodic environment in South Sudan for the year 2012 was 2.67 (with 95% CI). We further use this standardized model to predict the number of VL infected cases reported for the year 2013 in South Sudan [48]. The model predictions were highly comparable with the number of actual reported cases at the end of 2013 substantiating the predictive capability of the model.

Time dependent intervention strategies can be implemented to curtail a vector-borne disease on a finite time interval. Using optimal control analysis, we further investigated the effects of popular intervention strategies and their optimal combinations on infected human and vector populations in the model under periodic seasonal fluctuations, thereby depicting control in realistic situations. In this article, we consider three types of control, i.e. use of treated bednets, treatment of infectives and spray of insecticides. Tracking the time-dependent changes, it was observed that the combination of spray of insecticides & drug-based treatment of infected individuals (Strategy D) and the combination of treated bednet, spray of insecticides & drug-based treatment (Strategy A) performs well for the time period of intervention. To observe long term effect of control on disease spread, the effects of control strategies were observed on the basic reproduction number (*R*_0_) for entire period of intervention. This analysis indicated significant changes in the number of possible secondary infections from an infected individual, with respect to implementation of intervention strategies at different time points of the infection in population. Hence, it is important to determine the time point at which optimal control needs to be implemented. Also, complete elimination of the disease by any control strategy combination can be achieved only if the control strategies continue for long periods of time. These results pose a realistic view of visceral leishmaniasis disease spread, its control and its resurrection, once the control strategies are removed.

As opposed to the previous model on cutaneous leishmaniasis [35], we have considered the model specific to anthroponotic visceral leishmaniasis by incorporating the symptomatic, asymptomatic and transient infectious classes separately and implemented combinatory optimal control strategies and cost-effective analysis to propose an efficient elimination programme. Further, our predictions for visceral leishmaniasis are completely opposite to the model-based control strategies of cutaneous leishmaniasis [35], which predict complete elimination of the disease contradicting the endemicity observed in the real disease scenario after removal of a intervention strategy. Moreover, we have validated our study with real disease incidences and have shown the predictive capability of our model. From our study, the effect of the different strategies on *R*_0_ further indicated that the combination of treated bednets & spray of insecticides (Strategy C) performs the best to control disease, followed by strategies A & D which have comparable performance. Whereas, cost-effectiveness analysis using IAR and ICER, indicate that the combination of drug-based treatment of infective individuals and spray of insecticides (Strategy D) is the most optimal, cost-effective, and efficacious strategy followed by the combination of treated bednets and drug-based treatment control (Strategy B) to control disease dynamics. Strategy A also displays a high efficacy in eliminating the disease comparable to Strategy D, but is accompanied by a higher involved cost. Thus, we conclude that for cases where the severity of disease is low, it is favourable to choose both an efficacious and cost-effective strategy (Strategies B and D) whereas, when the intensity of the disease is high and the priority is to control the disease spread, strategies A or C, which are less-cost effective but immediately efficacious to control disease spread within a short span of time, can be applied. Thus, our model can be useful for decision makers, who are often faced by the challenge of resource allocation, to choose different control strategies with respect to the severity of the disease.

## Supporting information

S1 File Appendix. This file contains the mathematical analysis and calculations for the disease free equilibrium and its stability analysis, mathematical description of basic reproduction number (*R*_0_), existence and permanence of endemic periodic solution and its global stability from the model (Section A). Seasonality testing (Section B), parameter estimation and model sensitivity analysis (Section C). The file also contains the estimated model parameters and their description(Tables A and B) and initial values (Table C) of the variables considered in the model and Geweke’s Z-score for each parameter (Table D).
(PDF)

S1 Fig Estimation of model parameters. A) Posterior distribution of different parameters of the model B) Posterior distribution of *R*_0_ C)Trace Plots for all the parameters.
(TIF)

S2 Fig Plots of the PRCC of different parameters. The PRCC is calculated with respect to cumulative number of new VL cases with significant level 0.01, using 2000 samples.—A) Plot of PRCC values for the cumulative *I*_*C*_ compartment with time, B) Bar plot of PRCC of *R*_0_ for different model parameters.
(TIF)

S3 Fig Effect of optimal treatment to model variables. A) Number of susceptible and recovered individuals, B) Number of asymptomatic KA individuals, C) S3.B Fig (magnified, t = 90–100 days), D) Number of symptomatic KA and PKDL infected individuals, E) Number of infected vectors F) Control profile for optimal treatment policy.
(TIF)

S4 Fig Effect of the optimal treated bednet to model variables. A) Number of susceptible and recovered individuals, B) S4.B Fig (magnified, t = 90–100 days), C) Number of asymptomatic KA individuals, D) S4.C Fig (magnified, t = 90–100 days), E) Number of symptomatic KA and PKDL infected individuals, F) S4.E Fig (magnified, t = 90–100 days), G) Number of infected vectors, H) Control profile fr optimal treated bednet policy.
(TIF)

S5 Fig Effect of optimal spray of insecticide to model variables. A) Number of asymptomatic KA individuals, B) S5.A Fig (magnified, t = 90–100 days), C) Number of symptomatic KA and PKDL infected individuals, D) Number of infected vectors, E) Control profile for optimal spray of insecticide policy.
(TIF)

S6 Fig Total human population (*N*_*H*_) for the years 2012 and 2013 obtained from model simulations. (TIF)
