# Supplementary material for: Optimal combinations of control strategies and cost-effective analysis for visceral leishmaniasis disease transmission
Source: PLoS One. 2017 Feb 21;12(2):e0172465. doi: 10.1371/journal.pone.0172465 (PMC5319670; doi:10.1371/journal.pone.0172465)
Supplement: S1 File — This file contains the mathematical analysis and calculations for the disease free equilibrium and its stability analysis, mathematical description of basic reproduction number (R0), existence and permanence of endemic periodic solution and its global stability from the model (Section A). Seasonality testing (Section B), parameter estimation and model sensitivity analysis (Section C). The file also contains the estimated model parameters and their description(Tables A and B) and initial values (Table C) of the variables considered in the model and Geweke’s Z-score for each parameter (Table D). (PDF) [file pone.0172465.s001.pdf]

S1 File - Appendix

Section A:

Disease free equilibrium and its global stability

It is clear that the system (see "Model construction" in Methods Section) admits a unique disease free equilibrium $E_0 = (N_H, 0, 0, 0, 0, 0, N_R, 0, N_V, 0)$.

Let $(\mathbb{R}^n, \mathbb{R}^n_+)$ be an ordered n-dimensional Euclidean space with a norm $||.||$ and we assume that $\text{int}(\mathbb{R}^n_+) \neq \emptyset$. Let, $x,\ y \in \mathbb{R}^n$, we say $x \geqslant y$ if $x - y \in \mathbb{R}^n_+$; $x > y$ if $x - y \in \mathbb{R}^n_+ \backslash \{0\}$ and $x \gg y$ if $x - y \in \text{int}(\mathbb{R}^n_+)$.

The following lemma will be useful for proving global stability of E_0.

Lemma 1. *(Lemma 2.1 in [1]) Let $A(t)$ be a continuous, cooperative, irreducible, and ω-periodic $n \times n$ matrix function. Let $\Phi_{A(.)}(t)$ be the fundamental matrix of the linear non-autonomous differential equation $\dot{x} = A(t)x$, where x is a $n \times 1$ vector. Let $\mu = \frac{1}{\omega} \ln r(\Phi_{A(.)}(\omega))$, where, $r(\Phi_{A(.)}(\omega))$ is the spectral radius of the monodromy matrix $\Phi_{A(.)}(\omega)$. Then there exists a positive, ω-periodic function $v(t)$ such that $e^{\mu t} v(t)$ is a solution of $\dot{x} = A(t)x$.*

We assume that $x = (I_A, I_H, T_H, P_H, I_R, I_V)^T$, $\mathcal{F}(t, x)$ denotes the rate of new infection and $\mathcal{V}(t, x)$ denotes the transfer rate of individuals into or out of each infection compartment. Therefore, according to [2], we define matrices $F(t)$ and $V(t)$ as:

$$F(t) = \left(\frac{\partial \mathcal{F}_i(t, E_0)}{\partial x_j}\right)_{1 \leqslant i,j \leqslant 6} = \begin{pmatrix} 0 & 0 & 0 & 0 & 0 & a(t)b \\ 0 & 0 & 0 & 0 & 0 & 0 \\ 0 & 0 & 0 & 0 & 0 & 0 \\ 0 & 0 & 0 & 0 & 0 & 0 \\ 0 & 0 & 0 & 0 & 0 & a(t)b \\ \mu_1 a(t)cm & a(t)cm & 0 & a(t)cm & \mu_2 a(t)cn & 0 \end{pmatrix}$$

and,

$$V(t) = \left(\frac{\partial \mathcal{V}_i(t, E_0)}{\partial x_j}\right)_{1 \leqslant i,j \leqslant 6} = \begin{pmatrix} \gamma_h + \mu_H & 0 & 0 & 0 & 0 & 0 \\ -\rho_1 \gamma_h & \alpha_1 + \delta + \mu_H & 0 & 0 & 0 & 0 \\ 0 & -(1-\sigma)\alpha_1 & \delta_p + \mu_H & 0 & 0 & 0 \\ -\rho_2 \gamma_h & 0 & -\delta_p & \alpha_2 + \beta + \mu_H & 0 & 0 \\ 0 & 0 & 0 & 0 & \mu_R & 0 \\ 0 & 0 & 0 & 0 & 0 & \mu_V \end{pmatrix}.$$

Now , $F(t)$ is non-negative and $-V(t)$ is co-operative (off diagonal elements are non-negative). Thus, $F(t) - V(t)$ is continuous and co-operative and it can be easily shown that $F(t) - V(t)$ is irreducible. Therefore, lemma 1 is applicable to the system $\dot{X} = (F(t) - V(t))X$, where $X = (I_A, I_H, T_H, P_H, I_R, I_V)^T$.

The following result will be hold:

Proposition 1. *If* $r(\Phi_{F(.)-V(.)}(\omega)) < 1$ *then the disease free equilibrium* E_0 *is globally asymptotically stable.*

Proof. From our model system, we have the system of equations:

$$\begin{aligned}
I'_A &= a(t)bI_V\tfrac{S_H}{N_H} - (\gamma_h + \mu_H)I_A \\
I'_H &= \rho_1\gamma_h I_A - (\alpha_1 + \delta + \mu_H)I_H \\
T'_H &= (1-\sigma)\alpha_1 I_H - (\delta_p + \mu_H)T_H \\
P'_H &= \rho_2\gamma_h I_A + \delta_p T_H - (\alpha_2 + \beta + \mu_H)P_H \\
I'_R &= a(t)bI_V\tfrac{S_R}{N_R} - \mu_R I_R \\
I'_V &= \mu_1 a(t)cS_V\tfrac{I_A}{N_H} + a(t)cS_V\tfrac{I_H}{N_H} + a(t)cS_V\tfrac{P_H}{N_H} + \mu_2 a(t)cS_V\tfrac{I_R}{N_R} - \mu_V I_V
\end{aligned}$$

We observe that,

$$\frac{d}{dt}[I_A, I_H, T_H, P_H, I_R, I_V]^T \leqslant [F(t) - V(t)][I_A, I_H, T_H, P_H, I_R, I_V]^T.$$

Now, consider linear auxiliary system:

$$\frac{dX}{dt} = [F(t) - V(t)]X. \tag{0.1}$$

Applying lemma 1 there exists a ω-periodic function $\bar{X}(t)$ such that $X(t) = e^{st}\bar{X}(t)$ is a solution of the system (0.1), where $s = \frac{1}{\omega}\ln[r(\Phi_{F(.)-V(.)}(\omega))]$.

Now, by comparison principle [3], for any

$[(I_A(0), I_H(0), T_H(0), P_H(0), I_R(0), I_V(0)] \leqslant MX(0)$,

for some sufficiently large $M > 0$. We have $[I_A, I_H, T_H, P_H, I_R, I_V] \leqslant MX(t)$. Since, $r(\Phi_{F(.)-V(.)}(\omega)) < 1$, therefore, $X(t) \to 0$ as $t \to \infty$. Thus, $I_A(t) \to 0$, $I_H(t) \to 0$, $T_H(t) \to 0$, $P_H(t) \to 0$, $I_R(t) \to 0$ and $I_V(t) \to 0$ as $t \to \infty$.

Using theory of asymptomatic autonomous systems [4], we have $R_H(t) \to 0$, $S_H(t) \to 1$, $S_R(t) \to 1$, $S_V(t) \to 1$ as $t \to \infty$. Thus, E_0 is globally asymptotically stable if $r(\Phi_{F(.)-V(.)}(\omega)) < 1$.

□

Basic reproduction number

Let, for each $s \in \mathbb{R}$, the 6×6 matrix $Y(t, s), \forall t \geqslant s$, satisfies the ω-periodic system

$$\frac{dy}{dt} = -V(t)y. \tag{0.2}$$

and $Y(s, s) = I$, where I is the 6×6 identity matrix. Let, C_ω be the ordered Banach space of all ω-periodic functions from $\mathbb{R}$ to $\mathbb{R}^6$, which is equipped with maximum norm $||.||$ and the positive cone $C^+_\omega = \{\phi \in C_\omega : \phi(t) \geqslant 0, \forall t \in \mathbb{R}\}$. We define linear operator $L : C_\omega \to C_\omega$

$$(L\phi)(t) = \int_0^{\infty} Y(t, t-a)F(t-a)\phi(t-a)\mathrm{d}a, \quad \forall t \in \mathbb{R}, \;\; \phi \in C_\omega. \tag{0.3}$$

According to the definitions given by [2], L is known as the next infection operator. The spectral radius of L is defined as the basic reproduction number (R_0) with seasonality. R_0 is thus, defined as follows:

$$\bar{\bar{R}}_0 = r(L).$$

We further analyze our model (see "Model constriction" in Methods Section of main article) in terms of the threshold parameter R_0. We use following lemma to infer stability of E_0 in terms of R_0.

Lemma 2. *(Theorem 2.2 in [2]) The following statements are valid:*

1. $R_0 = 1$ *if and only if* $r(\Phi_{F(.)-V(.)}(\omega)) = 1$.
2. $R_0 > 1$ *if and only if* $r(\Phi_{F(.)-V(.)}(\omega)) > 1$.
3. $R_0 < 1$ *if and only if* $r(\Phi_{F(.)-V(.)}(\omega)) < 1$.

Therefore, from lemma 2 and proposition 1, we have the following result.

Proposition 2. *If $R_0 < 1$ then the disease free equilibrium E_0 of the model system is globally asymptotically stable.*

Existence and permanence of the endemic periodic solution

In this section, we study the existence and permanence of the endemic periodic state of the model.

Before going to the main result, we give following definitions from [5].

Definition 1. *The model system as defined is uniformly persistent if $\exists$ an $\eta > 0$ (depending only on parameter values not on initial condition) such that for any initial value*
$(S_H(0), I_A(0), I_H(0), T_H(0), P_H(0), R_H(0), S_R(0), I_R(0), S_V(0), I_V(0)) \in \mathbb{R}_+ \times$ *int*$(\mathbb{R}_+) \times$ *int*$(\mathbb{R}_+) \times$ *int*$(\mathbb{R}_+) \times$ *int*$(\mathbb{R}_+) \times$ *int*$(\mathbb{R}_+) \times \mathbb{R}_+ \times$ *int*$(\mathbb{R}_+) \times \mathbb{R}_+ \times$ *int*$(\mathbb{R}_+)$,

every solution $(S_H(t), I_A(t), I_H(t), T_H(t), P_H(t), R_H(t), S_R(t), I_R(t), S_V(t), I_V(t))$ *of the system satisfies*

$\lim_{t\to\infty} \inf S_H(t) \geqslant \eta$, $\lim_{t\to\infty} \inf I_A(t) \geqslant \eta$, $\lim_{t\to\infty} \inf I_H(t) \geqslant \eta$, $\lim_{t\to\infty} \inf T_H(t) \geqslant \eta$, $\lim_{t\to\infty} \inf P_H(t) \geqslant \eta$, $\lim_{t\to\infty} \inf R_H(t) \geqslant \eta$, $\lim_{t\to\infty} \inf S_R(t) \geqslant \eta$, $\lim_{t\to\infty} \inf I_R(t) \geqslant \eta$, $\lim_{t\to\infty} \inf S_V(t) \geqslant \eta$, $\lim_{t\to\infty} \inf I_V(t) \geqslant \eta$.

Definition 2. *The system is said to be permanent if there exists a compact region $\Omega_0 \in$ int(Ω) such that every solution of the system with initial condition*
$(S_H(0), I_A(0), I_H(0), T_H(0), P_H(0), R_H(0), S_R(0), I_R(0), S_V(0), I_V(0)) \in \mathbb{R}_+ \times$ *int*$(\mathbb{R}_+) \times$ *int*$(\mathbb{R}_+) \times$ *int*$(\mathbb{R}_+) \times$ *int*$(\mathbb{R}_+) \times$ *int*$(\mathbb{R}_+) \times \mathbb{R}_+ \times$ *int*$(\mathbb{R}_+) \times \mathbb{R}_+ \times$ *int*$(\mathbb{R}_+)$ *will eventually enter and remain in region Ω_0.*

Clearly, for a dissipative dynamical system proving permanence is equivalent to proving uniform persistence.

Consider following sets:

$X = \mathbb{R}_+^{10}$, $X_0 = \mathbb{R}_+ \times \text{int}(\mathbb{R}_+) \times \text{int}(\mathbb{R}_+) \times \text{int}(\mathbb{R}_+) \times \text{int}(\mathbb{R}_+) \times \text{int}(\mathbb{R}_+) \times \mathbb{R}_+ \times \text{int}(\mathbb{R}_+) \times \mathbb{R}_+ \times \text{int}(\mathbb{R}_+)$, $\partial X_0 = X \backslash X_0$.

Let, $f : X \to X$ be a continuous map and we define following set

$M_\partial = \{x \in \partial X_0 : f^n(x) \in \partial X_0, n \geqslant 0\}$.

Following lemma will be used to show uniform persistence of the considered model system

Lemma 3. *([5]) Assume that*

1. *$f(X_0) \subseteq X_0$ and f has a global attractor A.*
2. *There exists a finite sequence $\mathcal{M} = \{M_1, M_2, ..., M_k\}$ of disjoint, compact, and isolated invariant sets in ∂X_0 such that*
    - *$\Omega(M_\delta) = \cup_{x \in M_\partial} \omega(x) \subset \cup_{i=1}^k M_i$;*
    - *no subset of $\mathcal{M}$ forms a cycle in ∂X_0;*
    - *M_i is isolated in X;*
    - *$W^s(M_i) \cap X_0 = \emptyset$ for each $1 \leqslant i \leqslant k$*

Then there exists $\eta > 0$ such that $\liminf_{n\to\infty} d(f^n(x), \partial X_0) \geqslant \eta$ for all $x \in X_0$.

We claim the following result

Proposition 3. *If $R_0 > 1$ then the solutions of the system is uniformly persistent, and the system admits at least one positive ω-periodic solution.*

Proof. Consider the periodic semi-flow $T : \mathbb{R}_+^{10} \to \mathbb{R}_+^{10}$ associated with the model system defined by:

$T(t)x = u(t, x)$, $\forall x \in \mathbb{R}_+^{10}$.

Let P_1 be the associated Poincaré map defined as $P_1 := T(\omega)$. We first show that P_1 is uniformly persistent with respect to $(X_0, \partial X_0)$.

It is clear that the set X and X_0 are positively invariant for the model system. Now the bounded set Ω attracts every solution of the system and also Ω is compact. Thus the Poincaré map P_1 is point dissipative and compact on X. Therefore, it follows from Theorem 1.1.3 in [5] there is a global attractor A of P_1 that attracts each bounded set in X.

For our model system, the set M_∂ is defined as

$M_\partial = \{(S_H(0), I_A(0), I_H(0), T_H(0), P_H(0), R_H(0), S_R(0), I_R(0), S_V(0), I_v(0)) \in \partial X_0 :$
$P_1^n(S_H(0), I_A(0), I_H(0), T_H(0), P_H(0), R_H(0), S_R(0), I_R(0), S_V(0), I_v(0)) \in \partial X_0, \ \ \forall n \geqslant 0\}$.

We claim that, $M_\partial = \{(S_H, 0, 0, 0, 0, R_H, S_R, 0, S_V, 0) : S_H \geqslant 0, R_H \geqslant 0, S_R \geqslant 0, S_V \geqslant 0\}$. It is clear that, $\{(S_H, 0, 0, 0, 0, R_H, S_R, 0, S_V, 0) : S_H \geqslant 0, R_H \geqslant 0, S_R \geqslant 0, S_V \geqslant 0\} \subseteq M_\partial$.

Now, let $(S_H(0), I_A(0), I_H(0), T_H(0), P_H(0), R_H(0), S_R(0), I_R(0), S_V(0), I_v(0))$
$\in \partial X_0 \backslash \{(S_H, 0, 0, 0, 0, R_H, S_R, 0, S_V, 0) : S_H \geqslant 0, R_H \geqslant 0, S_R \geqslant 0, S_V \geqslant 0\}$.

If $I_A(0) = 0, I_H(0) = 0, T_H(0) = 0, P_H(0) = 0, I_R(0) = 0$, then we get $S_H(0) > 0, S_R(0) > 0, S_V(0) > 0, R_H(0) > 0, I_V(0) > 0$. From second equation of our model system we have,

$I'_A(0) > a(t)b\dfrac{I_V(0)S_H(0)}{N_H(0)} > 0.$

$I'_R(0) > a(t)b\dfrac{I_V(0)S_R(0)}{N_R(0)} > 0.$

Similarly, for other cases also.

Therefore, $(S_H(0), I_H(0), T_H(0), P_H(0), R_H(0), S_R(0), I_R(0), S_V(0), I_v(0)) \notin \partial X_0$ for all $0 < t \ll 1$. This implies that $M_\partial = \{(S_H, 0, 0, 0, 0, R_H, S_R, 0, S_V, 0) : S_H \geqslant 0, R_H \geqslant 0, S_R \geqslant 0, S_V \geqslant 0\}$.

Now, P_1 has a unique fixed point E_0 in M_∂. It is easy to show that $\{E_0\}$ is isolated in X and as E_0 is global attracting in M_∂ therefore we have, $\Omega(M_\partial) = \cup_{x \in M_\partial} \omega(x) \subseteq \{E_0\}$, where, $\omega(x)$ is the omega limit set of x. It is clear that no subset of $\{E_0\}$ can forms a cycle in ∂X_0.

Now, we shall show that, $W^s(E_0) \cap X_0 = \emptyset$, where, $W^s(E_0)$ is the stable set of E_0.

Let $x^0 = (S_H(0), I_A(0), I_H(0), T_H(0), P_H(0), R_H(0), S_R(0), I_R(0), S_V(0), I_v(0)) \in W^s(E_0) \cap X_0$.

Since $x^0 \in X_0$, therefore by continuity of solution with respect to initial conditions we have, for any $\epsilon \in (0, 1)$, there exists a $\delta > 0$ such that $\forall x^0 \in X_0$ satisfying $||x^0 - E_0|| < \delta$, implies $||u(t, x^0) - u(t, E_0)|| < \epsilon \ \forall t \in [0, \omega]$.

We claim that $\lim_{m \to \infty} \sup ||P_1^m(x^0) - E_0|| \geqslant \delta \ \forall x^0 \in X_0$. Suppose if possible $\exists x^0 \in X_0$ such that $\lim_{m \to \infty} \sup ||P_1^m(x^0) - E_0|| < \delta$. Without loss of generality, we can assume that $||P_1^m(x^0) - E_0|| < \delta$ for all $m \geqslant 0$. Therefore, we have $||u(t, P_1^m(x^0)) - u(t, E_0)|| < \epsilon$, $\forall t \in [0, \omega]$ and $\forall m \geqslant 0$.

For any, $t \geqslant 0$, let $t = m\omega + t_1$, where $t_1 \in [0, \omega]$ and $m = [\frac{t}{\omega}]$ is the greatest positive integer less than or equal to $\frac{t}{\omega}$.

Then we have, $||u(t, P_1^m(x^0)) - u(t, E_0)|| = ||u(t_1, P_1^m(x^0)) - u(t_1, E_0)|| < \epsilon$ for all $t \geqslant 0$.

Let, $(S_H(t), I_A(t), I_H(t), T_H(t), P_H(t), R_H(t), S_R(t), I_R(t), S_V(t), I_v(t)) = U(t, x^0)$. It follows from previous argument that, $1 - \epsilon < S_H(t) < 1 + \epsilon$, $1 - \epsilon < R_H(t) < 1 + \epsilon$, $1 - \epsilon < S_R(t) < 1 + \epsilon$, $1 - \epsilon < S_V(t) < 1 + \epsilon$, $0 < I_A(t) < \epsilon$, $0 < I_H(t) < \epsilon$, $0 < T_H(t) < \epsilon$, $0 < P_H(t) < \epsilon$, $0 < I_R(t) < \epsilon$, $0 < I_V(t) < \epsilon$, $\forall t \geqslant 0$.

From our model system we have:

$$\frac{d}{dt}[I_A, I_H, T_H, P_H, I_R, I_V]^T \geqslant [F(t) - V(t) - \epsilon M_\epsilon(t)][I_A, I_H, T_H, P_H, I_R, I_V]^T.$$

where, $M_\epsilon(t) = \begin{pmatrix} 0 & 0 & 0 & 0 & 0 & a(t)b\epsilon \\ 0 & 0 & 0 & 0 & 0 & 0 \\ 0 & 0 & 0 & 0 & 0 & \\ 0 & 0 & 0 & 0 & 0 & 0 \\ 0 & 0 & 0 & 0 & 0 & a(t)b\epsilon \\ \mu_1 a(t)cm\epsilon & a(t)cm\epsilon & 0 & a(t)cm\epsilon & \mu_2 a(t)cn\epsilon & 0 \end{pmatrix}$

Now, by lemma 2, we have if $R_0 > 1$ then $r(\Phi_{F(.)-V(.)}(\omega)) > 1$. Choosing $\epsilon > 0$ sufficiently small so that $r(\Phi_{F(.)-V(.)-\epsilon M_\epsilon(.)}(\omega)) > 1$. Thus by lemma 1 and standard comparison theorem [3] $\exists$ ω-periodic function $f(t)$ such that $x(t) \geqslant f(t)e^{s_1 t}$, where, $x(t) = [I_A, I_H, T_H, P_H, I_R, I_V]$ and

$$s_1 = \frac{1}{\omega}\ln r(\Phi_{F(.)-V(.)-\epsilon M_\epsilon(.)}(\omega)) > 0.$$

This implies as $t \to \infty$, $I_A(t) \to \infty$, $I_H(t) \to \infty$, $T_H(t) \to \infty$, $P_H(t) \to \infty$, $I_R(t) \to \infty$ and $I_V(t) \to \infty$. This is a contradiction as $\lim_{t\to\infty}\sup ||P_1^m(x^0) - E_0|| < \delta$. Therefore, we have, $\lim_{t\to\infty}\sup ||P_1^m(x^0) - E_0|| \geqslant \delta$ $\forall x^0 \in X_0$, which is again impossible as $x^0 \in W^s(E_0)$ (as $x \in W^s(E_0)$ implies $\lim_{t\to}\infty ||P_1^m(x^0) - E_0|| = 0$). Thus we have, $W^s(E_0) \cap X_0 = \emptyset$.

Therefore, by lemma 3 we have P_1 is uniformly persistent with respect to $(X_0, \partial X_0)$. Therefore, by Theorem 3.1.1 in [5] the periodic semi-flow T is uniformly persistent in X.

Thus, if $R_0 > 1$ then solution of the defined system is uniformly persistent.

Now the Poincarè map P_1 is point dissipative and compact and also P_1 is uniformly persistent with respect to $(X_0, \partial X_0)$. Now, X_0 is relatively open set in X and therefore, ∂X_0 is relatively closed set in X. Thus by Theorem 1.3.6 in [5], P_1 has a fixed point
$(\bar{S_H}(0), \bar{I}_A(0), \bar{I}_H(0), \bar{T}_H(0), \bar{P}_H(0), \bar{R_H}(0), \bar{S}_R(0), \bar{I}_R(0), \bar{S}_V(0), \bar{I}_V(0)) \in X_0$. Now we have, $\bar{I}_A(0) \in \text{int}(\mathbb{R}_+)$, $\bar{I}_H(0) \in \text{int}(\mathbb{R}_+)$, $\bar{T}_H(0) \in \text{int}(\mathbb{R}_+)$, $\bar{P}_H(0) \in \text{int}(\mathbb{R}_+)$, $\bar{I}_R(0) \in \text{int}(\mathbb{R}_+)$, $\bar{I}_V(0) \in \text{int}(\mathbb{R}_+)$. We need to show that, $\bar{S_H}(0) \in\text{int}(\mathbb{R}_+)$, $\bar{R_H}(0) \in\text{int}(\mathbb{R}_+)$, $\bar{S_R}(0) \in\text{int}(\mathbb{R}_+)$, $\bar{S_V}(0) \in\text{int}(\mathbb{R}_+)$. If not, suppose $\bar{S_H}(0) = 0$, then from model we have, $0 = -(\alpha_1 + \delta + \mu_H)\bar{I}_H(0) \Rightarrow \bar{I}_H(0) = 0$, a contradiction. Therefore, $\bar{S_H}(0) \in\text{int}(\mathbb{R}_+)$. Similarly, we can show that $\bar{R_H}(0) \in\text{int}(\mathbb{R}_+)$ $\bar{S_V}(0) \in \text{int}(\mathbb{R}_+)$ and $\bar{S_R}(0) \in \text{int}(\mathbb{R}_+)$.
Thus, $(\bar{S}_H(0), \bar{I}_A(0), \bar{I}_H(0), \bar{T}_H(0), \bar{P}_H(0), \bar{R}_H(0), \bar{S}_R(0), \bar{I}_R(0), \bar{S}_V(0), \bar{I}_V(0)) \in \text{int}(\mathbb{R}_+^{10})$.
Therefore, $(\bar{S_H}(t), \bar{I}_A(t), \bar{I}_H(t), \bar{T}_H(t), \bar{P}_H(t), \bar{R_H}(t), \bar{S}_R(t), \bar{I}_R(t), \bar{S}_V(t), \bar{I}_V(t))$
$= u(t, (\bar{S_H}(0), \bar{I}_A(0), \bar{I}_H(0), \bar{T}_H(0), \bar{P}_H(0), \bar{R_H}(0), \bar{S}_R(0), \bar{I}_R(0), \bar{S}_V(0), \bar{I}_V(0)))$ is the positive ω-periodic solution of the system. Thus if $R_0 > 1$ then system admits at least one positive periodic solution. □

Global stability of the positive periodic solution

We claim the following result

Proposition 4. *If $R_0 > 1$ then the model system has a unique positive ω-periodic solution which is globally asymptotically stable.*

Proof. If $R_0 > 1$ then we have by proposition 3, the model system admits a positive periodic solution We shall first show that $(\bar{S_H}(t), \bar{I}_A(t), \bar{I}_H(t), \bar{T}_H(t), \bar{P}_H(t), \bar{R_H}(t), \bar{S}_R(t), \bar{I}_R(t), \bar{S_V}(t), \bar{I_V}(t))$ is globally asymptotically stable if all the conditions of the proposition 4 are satisfied. We construct following Lyapunov function :
$L(t) = |S_H(t) - \bar{S_H}(t)| + |I_A(t) - \bar{I}_A(t)| + |I_H(t) - \bar{I}_H(t)| + |T_H(t) - \bar{T}_H(t)| + |P_H(t) - \bar{P}_H(t)| + |R_H(t) - \bar{R_H}(t)| + |S_R(t) - \bar{S}_R(t)| + |I_R(t) - \bar{I}_R(t)| + |S_V(t) - \bar{S_V}(t)| + |I_V(t) - \bar{I_V}(t)|$.
We use following formula, $|x|' = \text{sgn}(x)x'$,

to calculate the upper right-hand derivative (Dini's derivative) of $L(t)$.

Therefore, we have,

$$\begin{aligned} D^+L(t) \leqslant -|S_H - \bar{S_H}|(\mu_H) - |I_A - \bar{I}_A|(\mu_H) - |I_H - \bar{I}_H|(\mu_H + \delta) - |T_H - \bar{T}_H|(\mu_H) - |P_H - \bar{P}_H|(\mu_H) \\ -|R_H - \bar{R}_H|(\mu_H) - |S_R - \bar{S}_R|(\mu_R) - |I_R - \bar{I}_R|(\mu_R) - |S_V - \bar{S}_V|(\mu_V) - |I_V - \bar{I}_V|(\mu_V). \end{aligned} \tag{0.4}$$

Let, $K = \min\{\mu_H, \mu_R, \mu_V\}$. Therefore, $K > 0$.

Now,

$D^+L(t) \leqslant -K(|S_H - \bar{S_H}| + |I_A - \bar{I}_A| + |I_H - \bar{I}_H| + |T_H - \bar{T}_H| + |P_H - \bar{P}_H| + |R_H - \bar{R}_H| + |S_R - \bar{S}_R| + |I_R - \bar{I}_R| + |S_V - \bar{S}_V| + |I_V - \bar{I}_V|)$.

Which implies L is non-increasing on $[0, +\infty)$. Integrating the above inequality from 0 to t we have,

$$\begin{aligned} L(t) + K \textstyle\int_0^t(|S_H - \bar{S_H}| + |I_A - \bar{I}_A| + |I_H - \bar{I}_H| + |T_H - \bar{T}_H| + |P_H - \bar{P}_H| \\ +|R_H - \bar{R}_H| + |S_R - \bar{S}_R| + |I_R - \bar{I}_R| + |S_V - \bar{S}_V| + |I_V - \bar{I}_V|) \leqslant L(0) < \infty, \qquad \forall t \geqslant 0. \end{aligned} \tag{0.5}$$

Thus we have, $\lim_{t\to\infty} L(t) = 0$. Therefore it follows that

$\lim_{t\to\infty} |S_H - \bar{S_H}| = 0$, $\lim_{t\to\infty} |I_A - \bar{I}_A| = 0$, $\lim_{t\to\infty} |I_H - \bar{I}_H| = 0$, $\lim_{t\to\infty} |T_H - \bar{T}_H| = 0$, $\lim_{t\to\infty} |P_H - \bar{P}_H| = 0$, $\lim_{t\to\infty} |R_H - \bar{R_H}| = 0$, $\lim_{t\to\infty} |S_R - \bar{S}_R| = 0$, $\lim_{t\to\infty} |I_R - \bar{I}_R| = 0$, $\lim_{t\to\infty} |S_V - \bar{S}_V| = 0$, $\lim_{t\to\infty} |I_V - \bar{I}_V| = 0$.

Thus, $(\bar{S_H}(t), \bar{I}_A(t), \bar{I}_H(t), \bar{T}_H(t), \bar{P}_H(t), \bar{R_H}(t), \bar{S}_R(t), \bar{I}_R(t), \bar{S_V}(t), \bar{I_V}(t))$ is globally asymptotically stable.

Now we shall show that there exist unique ω-periodic solution of the model system. For any two ω- periodic solutions $(\bar{S_H}(t), \bar{I}_A(t), \bar{I}_H(t), \bar{T}_H(t), \bar{P}_H(t), \bar{R_H}(t), \bar{S}_R(t), \bar{I}_R(t), \bar{S_V}(t), \bar{I_V}(t))$ and $(\bar{\bar{S_H}}(t), \bar{\bar{I}}_A(t), \bar{\bar{I}}_H(t), \bar{\bar{T}}_H(t), \bar{\bar{P}}_H(t), \bar{\bar{R_H}}(t), \bar{\bar{S}}_R(t), \bar{\bar{I}}_R(t), \bar{\bar{S_V}}(t), \bar{\bar{I_V}}(t))$ of the model system, we claim that $\bar{S_H}(t) = \bar{\bar{S_H}}(t)$; $\bar{I}_A(t) = \bar{\bar{I}}_A(t)$; $\bar{I}_H(t) = \bar{\bar{I}}_H(t)$; $\bar{T}_H(t) = \bar{\bar{T}}_H(t)$ $\bar{P}_H(t) = \bar{\bar{P}}_H(t)$; $\bar{R_H}(t) = \bar{\bar{R_H}}(t)$; $\bar{S}_R(t) = \bar{\bar{S}}_R(t)$; $\bar{I}_R(t) = \bar{\bar{I}}_R(t)$; $\bar{S}_V(t) = \bar{\bar{S}}_V(t)$; and $\bar{I}_V(t) = \bar{\bar{I}}_V(t)$, for all $t \in [0, \omega]$. If not, then there must be at least one $\eta \in [0, \omega]$ such that $\bar{S_H}(\eta) \neq \bar{\bar{S_H}}(\eta)$ *i.e.* $|\bar{S_H}(\eta) - \bar{\bar{S_H}}(\eta)| = \varepsilon > 0$.

Thus we can get

$$\begin{aligned} \varepsilon &= \lim_{n\to\infty} |\bar{S_H}(\eta + n\varpi) - \bar{\bar{S_H}}(\eta + n\varpi)| \\ &= \lim_{t\to\infty} |\bar{S_H}(t) - \bar{\bar{S_H}}(t)| > 0 \end{aligned}$$

Which is a contradiction to the fact that $(\bar{S_H}(t), \bar{I}_A(t), \bar{I}_H(t), \bar{T}_H(t), \bar{P}_H(t), \bar{R_H}(t), \bar{S}_R(t), \bar{I}_R(t), \bar{S_V}(t), \bar{I_V}(t))$ is globally asymptotically stable. Therefore $\bar{S_H}(t) = \bar{\bar{S_H}}(t)$, $\forall t \in [0, \omega]$ and similarly for other cases also. Therefore, $\bar{S_H}(t) = \bar{\bar{S_H}}(t)$; $\bar{I}_A(t) = \bar{\bar{I}}_A(t)$; $\bar{I}_H(t) = \bar{\bar{I}}_H(t)$; $\bar{T}_H(t) = \bar{\bar{T}}_H(t)$; $\bar{P}_H(t) = \bar{\bar{P}}_H(t)$ $\bar{R_H}(t) = \bar{\bar{R_H}}(t)$; $\bar{S}_R(t) = \bar{\bar{S}}_R(t)$; $\bar{I}_R(t) = \bar{\bar{I}}_R(t)$; $\bar{S}_V(t) = \bar{\bar{S}}_V(t)$; and $\bar{I}_V(t) = \bar{\bar{I}}_V(t)$, for all $t \in [0, \omega]$.

Thus if all conditions of the proposition 4 are satisfied and if $R_0 > 1$, then system has an unique positive ω-periodic solution which is globally asymptotically stable.

The inequalities in Proposition 3 and 4 indicate the strong dependence on sandfly parameters like periodic per-capita biting rate (a), periodic sandfly to human transmission rate (b), and transmission probability for

sandfly infection (c). This indicates an important role of the sandfly in controlling endemic nature of the disease.

Optimal control

The necessary conditions that an optimal control must satisfy, come from the Pontryagin's Maximum Principle. The Hamiltonian H, with respect to control functions u_1, u_2, u_3 and u_4 as defined in the main article can be written as:

$$\begin{aligned} H &= (A_1 I_A(t) + A I_H(t) + A_2 P_H(t) + \frac{1}{2} B u_1^2 + \frac{1}{2} C u_2^2 + \frac{1}{2} D u_3^2 + \frac{1}{2} E u_4^2) \\ &+ \sum_{i=1}^{10} \lambda_i g_i + \lambda_{C_{VL}} \{c_b u_1 S_H + c_{t1} u_2 I_H + c_{t2} u_3 P_H + c_v u_4 (S_V + I_V)\} \end{aligned}$$

where g_i is the right hand side of the differential equation of the ith state variable and λ_i are the adjoint variables. The associated adjoint functions are given by:

$$\frac{\partial \lambda_i}{\partial t} = f_i, i = 1, ..., 10 \; and \; \frac{\partial \lambda_{C_{VL}}}{\partial t} = 0.$$

where

$$
\begin{aligned}
f_1 &= -c_b u_1(t)\lambda_{C_{VL}} + (a(t)b\frac{I_V}{N_H}(1-u_1(t))(1-\frac{S_H}{N_H}))(\lambda_1-\lambda_2) + \mu_H\lambda_1 \\
&- (\lambda_9-\lambda_{10})(1-u_1(t))(\mu_1 a(t)cS_V\frac{I_A}{{N_H}^2} + a(t)cS_V\frac{I_H}{{N_H}^2} + a(t)cS_V\frac{P_H}{{N_H}^2}) \\
f_2 &= -A_1 + \lambda_2(\mu_H+\gamma_h) - a(t)b\frac{I_V S_H}{{N_H}^2}(1-u_1(t))(\lambda_1-\lambda_2) \\
&- \gamma_h(\rho_1\lambda_3+\rho_2\lambda_5+\rho_3\lambda_6) + (\mu_1 a(t)c\frac{S_V}{N_H})(\lambda_9-\lambda_{10})(1-u_1(t)) \\
&- (\lambda_9-\lambda_{10})(1-u_1(t))(\mu_1 a(t)cS_V\frac{I_A}{{N_H}^2} + a(t)cS_V\frac{I_H}{{N_H}^2} + a(t)cS_V\frac{P_H}{{N_H}^2}) \\
f_3 &= -A + (u_2(t)+\mu_H+\delta)\lambda_3 - u_2(t)((1-\sigma)\lambda_4+\sigma\lambda_5) \\
&+ a(t)c\frac{S_V}{N_H}(\lambda_9-\lambda_{10})(1-u_1(t)) - (\lambda_9-\lambda_{10})(1-u_1(t))(\mu_1 a(t)cS_V\frac{I_A}{{N_H}^2} \\
&+ a(t)cS_V\frac{I_H}{{N_H}^2} + a(t)cS_V\frac{P_H}{{N_H}^2}) - c_{t1}u_2(t)\lambda_{C_{VL}} \\
f_4 &= (\delta_p+\mu_H)\lambda_4 - \delta_p\lambda_5 - (\lambda_9-\lambda_{10})(1-u_1(t))(\mu_1 a(t)cS_V\frac{I_A}{{N_H}^2} + a(t)cS_V\frac{I_H}{{N_H}^2} \\
&+ a(t)cS_V\frac{P_H}{{N_H}^2}) \\
f_5 &= -A_2 + (u_2(t)+\beta+\mu_H)\lambda_5 - (u_2(t)+\beta)\lambda_6 + a(t)c\frac{S_V}{N_H}(\lambda_9-\lambda_{10})(1-u_1(t)) \\
&- (\lambda_9-\lambda_{10})(1-u_1(t))(\mu_1 a(t)cS_V\frac{I_A}{{N_H}^2} + a(t)cS_V\frac{I_H}{{N_H}^2} + a(t)cS_V\frac{P_H}{{N_H}^2}) \\
&- c_{t2}u_3(t)\lambda_{C_{VL}} \\
f_6 &= (\mu_H+\rho_r)\lambda_6 - \rho_r\lambda_1 - (\lambda_9-\lambda_{10})(1-u_1(t))(\mu_1 a(t)cS_V\frac{I_A}{{N_H}^2} + a(t)cS_V\frac{I_H}{{N_H}^2} \\
&+ a(t)cS_V\frac{P_H}{{N_H}^2}) \\
f_7 &= a(t)b\frac{I_V}{N_R}(1-\frac{S_R}{N_R})(\lambda_7-\lambda_8) + \mu_R\lambda_7 - \mu_2 a(t)c\frac{S_V I_R}{{N_R}^2}(\lambda_9-\lambda_{10}) \\
f_8 &= \mu_R\lambda_8 - a(t)b\frac{I_V S_R}{{N_R}^2}(\lambda_7-\lambda_8) - \mu_2 a(t)c\frac{S_V I_R}{{N_R}^2}(\lambda_9-\lambda_{10}) \\
&+ \mu_2 a(t)c\frac{S_V}{N_R}(\lambda_9-\lambda_{10}) \\
f_9 &= \{(\mu_1 a(t)c\frac{I_A}{N_H} + a(t)c\frac{P_H}{N_H} + a(t)c\frac{I_H}{N_H})(1-u_1(t)) + \mu_2 a(t)c\frac{I_R}{N_R}\}(\lambda_9-\lambda_{10}) \\
&+ (\mu_V+u_4(t))\lambda_9 - c_v u_4(t)\lambda_{C_{VL}} \\
f_{10} &= a(t)b\frac{S_H}{N_H}(1-u_1(t))(\lambda_1-\lambda_2) + a(t)b\frac{S_R}{N_R}(\lambda_7-\lambda_8) + (\mu_V+u_4(t))\lambda_{10} \\
&- c_v u_4(t)\lambda_{C_{VL}}
\end{aligned}
$$

Section B:

Seasonality

To justify whether the existence of any kind of seasonal forcing influence the number of VL incidence in South Sudan, a suitable statistical testing procedure is very much needed. The monthly data from 1st January, 2012 to 31st December, 2012 is taken into account for this purpose. We follow the test procedure suggested by Walter and Elwood[17].

The entire span of 1 year is divided into 12 classes, corresponding to the 12 months of an year and the total number of VL cases in month i (i= 1,2,...,12) is denoted by N_i . The probability that any one event belongs to i^{th} class is P_i , where

$$P_i = \frac{q_i(1+\alpha s_i+\beta c_i)}{\sum_{i=1}^{12} q_i(1+\alpha s_i+\beta c_i)},$$

where, q_i denotes the frequency for class i under the null hypothesis, $s_i = \sin(2\pi/12)$, $c_i = \cos(2\pi/12)$, α and β are the parameters of the above model. $H_0 : \alpha = \beta = 0$ indicates the absence of seasonality and H_1 : $\alpha \neq 0$ or $\beta \neq 0$ indicate the seasonality in VL incidence.
The test statistics for testing H_0 is of the form

$$R = (\frac{\overline{x}-\mu_{\overline{x}}}{\sigma_{\overline{x}}})^2 + (\frac{\overline{y}-\mu_{\overline{y}}}{\sigma_{\overline{y}}})^2.$$

The test statistic R is asymptotically distributed as λ^2 with 2 degrees of freedom. Here, $\overline{x}, \mu_{\overline{x}}$ and $\sigma_{\overline{x}}$ are defined as per Walter and Elwood[17].

In our case, test statistic R=18.51. The seasonality result is found to be positive with a significance level of 0.05, suggesting a significant influence of seasonality in VL disease dynamics.

Section C:

Parameter estimation

The model parameters were estimated using the number of VL cases reported in South Sudan for the year 2012 [6] (see "Model Calibration" in Methods section of main article). The estimated model parameters, including human, reservoir and sandfly demographic parameters, for South Sudan in 2012 are given in Tables A and B. The initial values used for the numerical simulations are reported in Table C. Plots for the posterior distributions of the estimated parameters, including human, reservoir and sandfly demographic parameters of the model are given in Fig S1.A. Observing the estimated initial values of different infected human compartments (Table C) in the South Sudan indicate the fact that the epidemic had already spread within the population at the time of the recorded data (January, 2012). Our model fitting procedure estimated that the amplitude of the monthly sandfly biting rate is 0.01 (with 95% CI). The estimated value of transmission probability measuring human susceptibility to infection was found to be on average 0.10 (with 95% CI).

The basic reproductive number (R_0), the expected number of secondary cases produced by a single infection in a completely susceptible population, was calculated for our model and the estimated value of the basic reproduction number in periodic environment is 2.67 (with 95% CI). The distribution of R_0 is given in Fig. S1.B. Trace plot of all the parameters are given in Fig S1.C. Parameter trace plots gives an idea about the stationary state of the Markov chain. In our model, we observe that the chain has reached to it's stationary distribution as the mean and the variance of the trace plot keeps relatively constant. Geweke's Z-scores (Table D) were used to ensure the chain convergence.

Sensitivity analysis - Results

Sensitivity analysis was performed for 2000 random parameter samples to conclude about the system (see "Model Sensitivity Analysis" in Methods section of main article). S2.A Fig describes that the most influential parameter with respect to I_C is ρ_1, ρ_1 has positive correlation with I_C. Due to the importance of R_0, we performed sensitivity analysis in R_0 (S2.B Fig) as well. Transmission probability (b) and biting rate (a_0) are the most important parameters in determining the magnitude of R_0. A positive PRCC value indicates that an increase in that parameter leads to an increase in R_0, while a negative value shows that increasing that parameter decreases R_0. $k_1, k_2, b, a_0, \gamma_H, \mu_1, \mu_2$ are positively correlated while $\alpha_1, \rho_1, \rho_2, \delta_r$ are negatively correlated with R_0.

References

1. Zhang F., Zhao, X.A periodic epidemic model in a patchy environment. J. Math. Anal. App. 2007; 325(1):496-516.
2. Wang W., Zhao X. Threshold dynamics for compartmental epidemic models in periodic environments. J. Dyn. Diff. Eq. 2008; 20(3):699-717.
3. Smith H., Waltman P. The Theory of the Chemostat: Dynamics of Microbial Competition. Cambridge Univ Press. 1995.
4. Thieme H. Convergence results and Poincare-Bendixson trichotomy for asymptomatically autonomous differential equations. J Math Biol. 1992; 30(7):755- 763.
5. Zhao X. Q. Dynamical Systems in Population Biology. Springer-Verlag, New York. 2003.
6. Abubakar A., Ruiz-Postigo J. A., Pita J., Lado M., Ben-Ismail R., Argaw D. et al. Visceral leishmaniasis outbreak in south Sudan 20092012: epidemiological assessment and impact of a multisectoral response. PLoS Negl Trop Dis. 2014; 8(3):e2720.

7. Sardar T., Rana S., Bhattacharya S., Al-Khaled K., Chattopadhyay J. A generic model for a single strain mosquito-transmitted disease with memory on the host and the vector. Mathematical Biosciences 2015; 263:18-36.

8. The World Bank, 2012. http://data.worldbank.org/country/south-sudan/.

9. Stauch A., Sarkar R., Picado A., Ostyn B., Sundar S., Rijal S. et al. Visceral leishmaniasis in the Indian subcontinent: modelling epidemiology and control. PLoS Negl Trop Dis. 2011; 5(11):e1405.

10. Dye C. The logic of visceral leishmaniasis control. Am J Trop Med Hyg. 1996; 55(2):125-130.

11. Sundar S., Lockwood D., Agrawal G., Rai M., Makharia M., Murray H. Treatment of Indian visceral leishmaniasis with single or daily infusions of low dose liposomal Amphotericin B: randomised trial commentary: cost and resistance remain issues. Bmj 2001; 323(7310):419-422.

12. Gasim S., Elhassan A., Kharazmi A., Khalil E., Ismail A., Theander T. The development of post kala azar dermal leishmaniasis (PKDL) is associated with acquisition of *Leishmania* reactivity by peripheral blood mononuclear cells (pbmc). Clin Exp Immunol 2000; 119(3):523-529.

13. Chapman L.A., Dyson, L., Courtenay, O., Chowdhury, R., Bern, C.,Medley, G.F., Hollingsworth , T. D. Quantification of the natural history of visceral leishmaniasis and consequences for control. Parasites and vectors 2015, 8(1):113.

14. Okusun K. O., Rachid O., Marcus N. Optimal control strategies and cost-effectiveness analysis of a malaria model. BioSystems 2013; 111:83-101.

15. Lee B., Bacon K., Shah M., Kitchen S., Connor D., Slayton R. The economic value of a visceral leishmaniasis vaccine in Bihar state, India. Am J Trop Med Hyg. 2012; 86(3):417-425.

16. Ozaki M., Islam S., Rahman K., Rahman A., Luby S., Bern C. Economic Consequences of Post-Kala-Azar Dermal Leishmaniasis in a Rural Bangladeshi Community. Am J Trop Med Hyg. 2011; 85(3):528–534.

17. Walter S. D., Elwood J.M. A test for seasonality of events with a variable population at risk. Br J Prev Soc Med. 1975; 29:18-21.

Table A: Estimated model parameters R_0 is not a parameter, but rather a function of the other parameters.

Parameter	Description	Value	Range	Source
Λ_H	Constant recruitment rate of Human	$\mu_H * N_H(0)\ day^{-1}$	-	[7]
μ_H	death rate of Human	$\frac{1}{54.72}\ year^{-1}$	-	[8]
Λ_R	Constant recruitment rate of Reservoir	$\mu_R * N_R(0)\ day^{-1}$	-	[9]
μ_R	Death rate of Reservoir	$0.0017\ day^{-1}$	-	[10]
Λ_V	Constant recruitment rate of Reservoir	$\mu_V * N_V(0)\ day^{-1}$	-	[9]
μ_V	Death rate of Sandfly	$0.0668\ day^{-1}$	-	[9]
δ	Death rate due to VL	$0.011\ day^{-1}$	-	[11]
$\frac{1}{\delta_p}$	Duration until relapse to PKDL stage	630 days	-	[9]
α_2	Treatment rate in PKDL stage	$0.033\ day^{-1}$	-	[12]
a_0	Average biting rate of sandflies	$0.20\ day^{-1}$	[0.15, 0.3]	[9]
ρ_r	Rate of loss of disease induced immunity	$0.002\ day^{-1}$	[0.001, 0.006]	[9]
α_1	Treatment rate of VL in symptomatic KA stage	$0.03\ day^{-1}$	[0.01, 0.04]	[9]
β	Natural recovery rate of human from PKDL stage	$0.005\ day^{-1}$	[0.001,0.005]	[13]
ρ_1	Fraction of asymptomatically infected hosts who will develop symptomatic KA	0.01	[0.01, 0.02]	[9]
ρ_2	Fraction of asymptomatically infected hosts who will develop PKDL	0.0001	[0.0001, 0.0002]	[9]
ρ_3	Fraction of asymptomatically infected hosts who will recover	$1 - \rho_1 - \rho_2$	-	-
γ_h	Sojourn time in the asymptomatic KA stage	$0.06\ day^{-1}$	[0.02, 0.06]	[9]
σ	Proportion of patients from symptomatic KA stage to recovered human stage	0.84	[0.80, 0.90]	[9]
δ_r	The amplitude of seasonality	0.01	[0.01,0.1]	Estimated, 95% CI
b	Transmission probability of VL in human and reservoir population	0.10	[0.04,0.20]	Estimated, 95% CI
c	Transmission probability of VL in sandfly population	0.10	[0.01, 0.20]	Estimated,95% CI

Parameter	Description	Value	Range	Source
k_1	Total number of reservoir per human	0.73	[0.10, 1.5]	Estimated, 95% CI
k_2	Total number of sandfly per human	1.62	[0.10, 3.2]	Estimated, 95% CI
μ_1	Infection probability of sandfly for biting humans in asymptomatic KA stage	0.01	[0.01, 0.05]	[9]
μ_2	Infection probability of sandfly for biting reservoir	1	-	[9]
R_0	Basic reproduction number	2.67	[1.88, 3.67]	Estimated, 95% CI

Estimated parameters are given in the format [estimate(95% CI)]. $N_H(0)$ is the initial total human population size, $N_R(0)$ is the initial total reservoir population size and $N_V(0)$ is the initial total sandfly population size.

Table B: Computational parameters Values

Computational parameter	Symbol	Value	Source
Final time	T	100 days	Assumed
Upper bound for controls u_1	b_1	1	Assumed
Lower bound for controls u_1	a_1	0	Assumed
Upper bound for controls u_2	b_2	1	Assumed
Lower bound for controls u_2	a_2	0	Assumed
Upper bound for controls u_3	b_3	1	Assumed
Lower bound for controls u_4	a_4	0	Assumed
Upper bound for controls u_4	b_4	1	Assumed
Lower bound for controls u_2	a_2	0	Assumed
Weight factor associated with u_1	B	1	Assumed
Weight factor associated with u_2	C	1	Assumed
Weight factor associated with u_3	D	1	Assumed
Weight factor associated with u_4	E	1	Assumed
Weight factor associated with I_A	A_1	1	Assumed
Weight factor associated with I_H	A	1	Assumed
Weight factor associated with P_H	A_2	1	Assumed
Per person unit cost of bed nets	c_b	$ 4	[14]
Per person unit cost of treatment and care for symptomatic KA patients	c_{t1}	$ 386.42	[15]
Per person unit cost of treatment for PKDL patients	c_{t2}	$ 56	[16]
Per area cost of insecticides spray	c_v	$ 1.50	[14]
Discount rate	σ_1	4/365	Assumed

$'\sigma_1'$ means discount rate (the discount rate applied to future years). The unit of time becomes a less natural and more synthetic measure if the future is discounted, as in all these analysis. The discounting procedure reflects inherent uncertainty about the future. Final time means interventions are applied for T days.

Table C: Demographic parameters description, values and Posterior summary.

Demographic Parameter	Description	Lower bound	Upper bound	Source/ Estimated value
$N_H(0)$	Initial total human population	1.098×10^7	1.098×10^7	[8]
$S_H(0)$	Initial susceptible human population	$N_H(0) * 0.80$	$N_H(0) * 0.90$	8.93×10^6
$I_H(0)$	Initial symptomatic KA infected human population	C(0)*0.80	$C(0) * 0.88$	884
$P_H(0)$	Initial PKDL infected human population	C(0)*0.08	$C(0) * 0.12$	27
$R_H(0)$	Initial size of human population who are immune to VL	$N_H(0) * 0.08$	$N_H(0) * 0.10$	1.26×10^6
$I_V(0)$	Initial size of infected sandfly population	0	$N_V(0) * 0.0002$	2258
$I_R(0)$	Initial size of infected reservoir population	0	$N_R(0) * 0.0004$	1879

$N_H(0)$ is the initial total human population size, $N_R(0)$ is the initial total reservoir population size, $N_V(0)$ is the initial total sandfly population size and $C(0)$ is the first data point from Fig 4 of the main article. All estimated demographic parameters of the model are given in the format [estimate (95% CI)].

Table D: Geweke's Z-score of each parameter

Parameter	Geweke's Z-score
δ_r	0.99115
b	0.97081
k_1	0.89755
k_2	0.99092
c	0.94368
$S_{H(0)}$	0.99623
$I_{H(0)}$	0.99025
$P_{H(0)}$	0.80434
$R_{H(0)}$	0.96894
$I_{R(0)}$	0.81282
$I_{V(0)}$	0.9926
