# Supplementary figures and images for: Optimal combinations of control strategies and cost-effective analysis for visceral leishmaniasis disease transmission
Source: PLoS One. 2017 Feb 21;12(2):e0172465. doi: 10.1371/journal.pone.0172465 (PMC5319670; doi:10.1371/journal.pone.0172465)

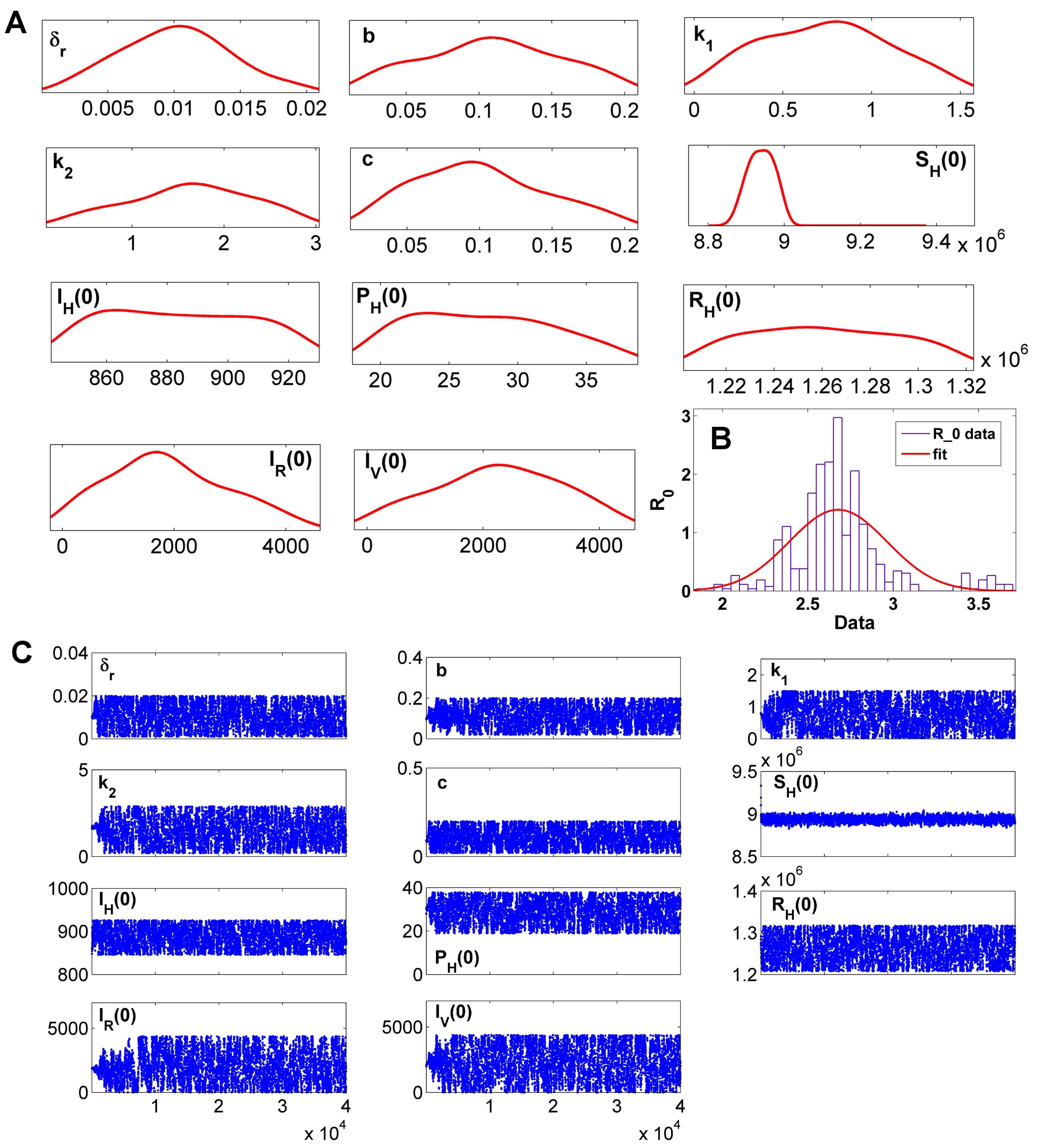

Supplement: S1 Fig — A) Posterior distribution of different parameters of the model B) Posterior distribution of R0 C)Trace Plots for all the parameters. (TIF) [file pone.0172465.s002.tif]

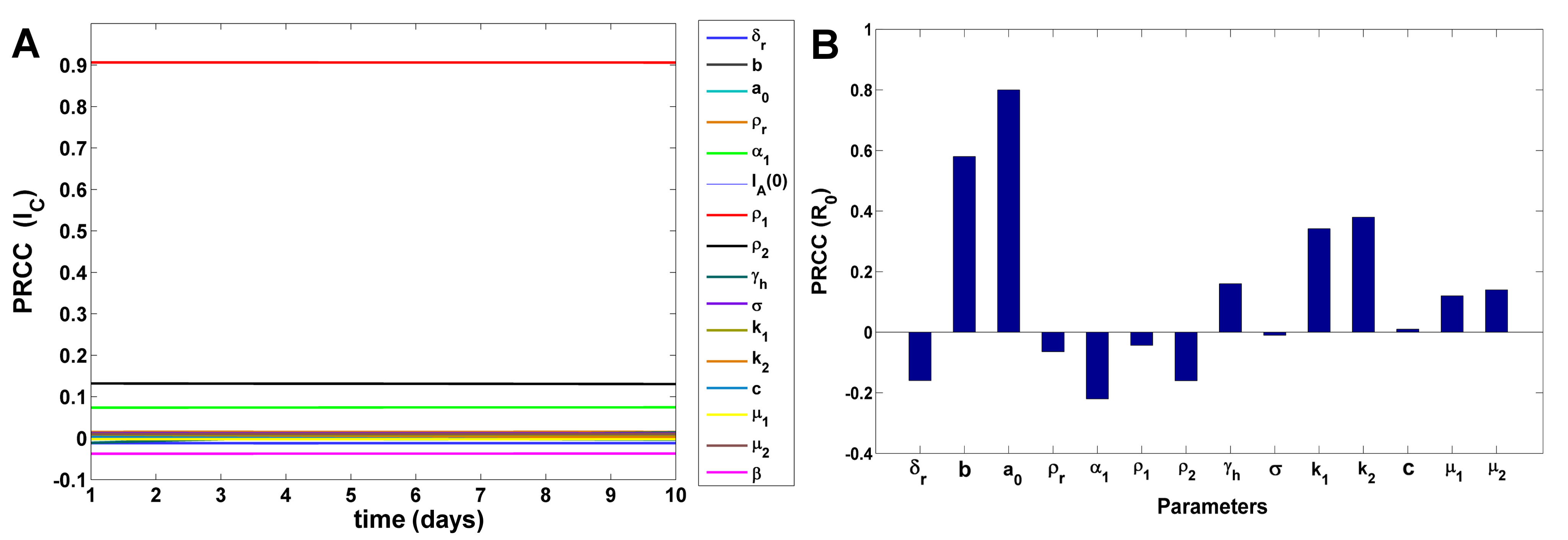

Supplement: S2 Fig — The PRCC is calculated with respect to cumulative number of new VL cases with significant level 0.01, using 2000 samples.—A) Plot of PRCC values for the cumulative IC compartment with time, B) Bar plot of PRCC of R0 for different model parameters. (TIF) [file pone.0172465.s003.tif]

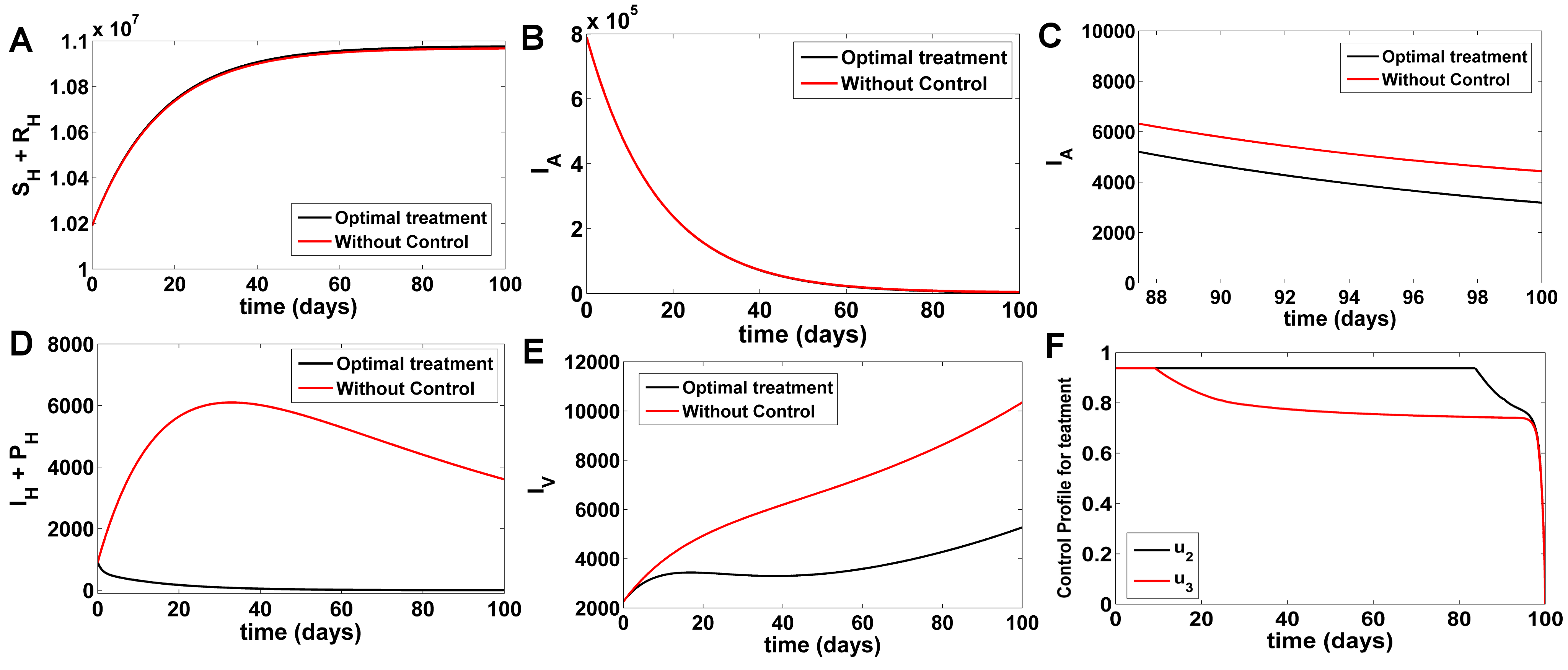

Supplement: S3 Fig — A) Number of susceptible and recovered individuals, B) Number of asymptomatic KA individuals, C) S3.B Fig (magnified, t = 90–100 days), D) Number of symptomatic KA and PKDL infected individuals, E) Number of infected vectors F) Control profile for optimal treatment policy. (TIF) [file pone.0172465.s004.tif]

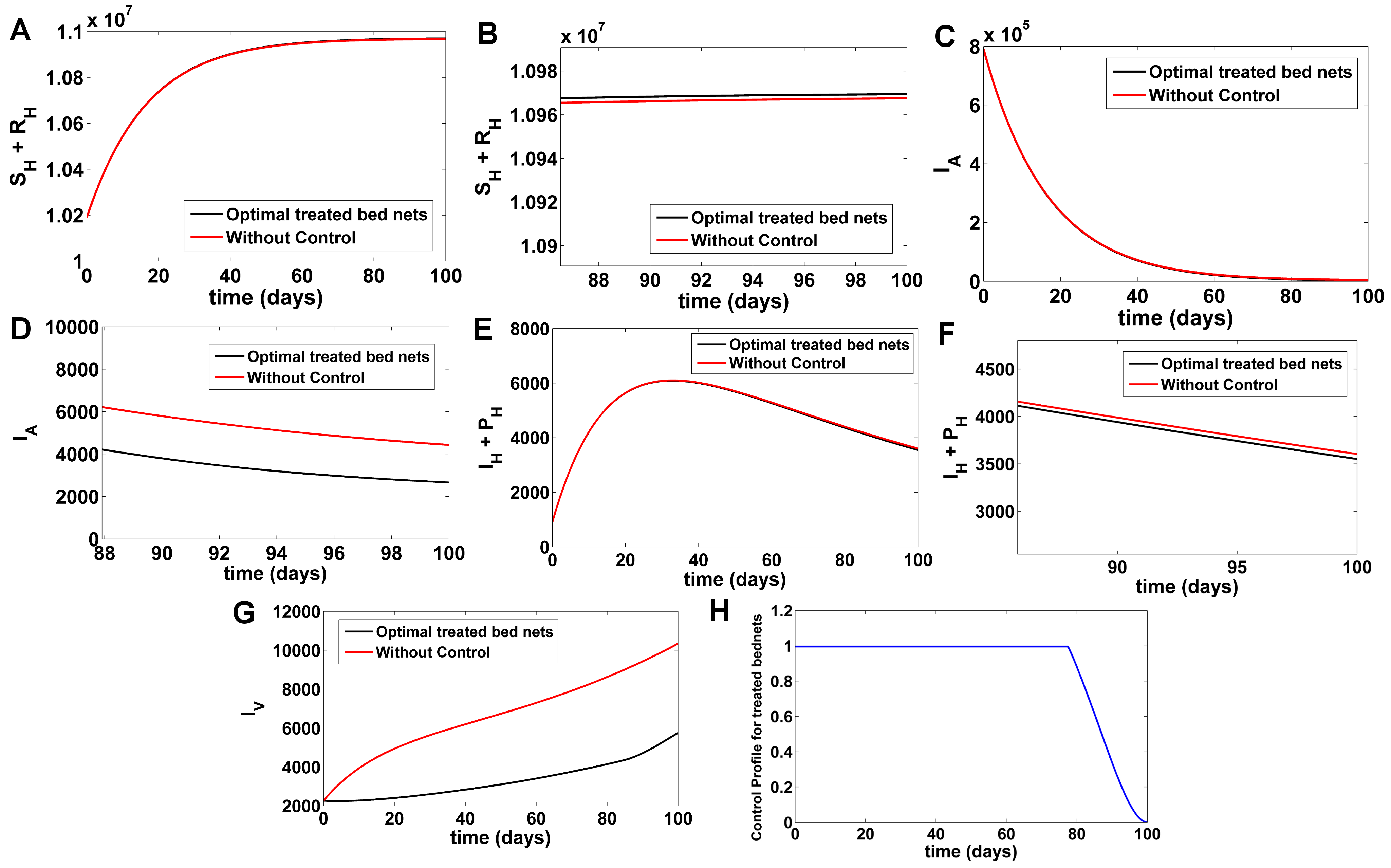

Supplement: S4 Fig — A) Number of susceptible and recovered individuals, B) S4.B Fig (magnified, t = 90–100 days), C) Number of asymptomatic KA individuals, D) S4.C Fig (magnified, t = 90–100 days), E) Number of symptomatic KA and PKDL infected individuals, F) S4.E Fig (magnified, t = 90–100 days), G) Number of infected vectors, H) Control profile fr optimal treated bednet policy. (TIF) [file pone.0172465.s005.tif]

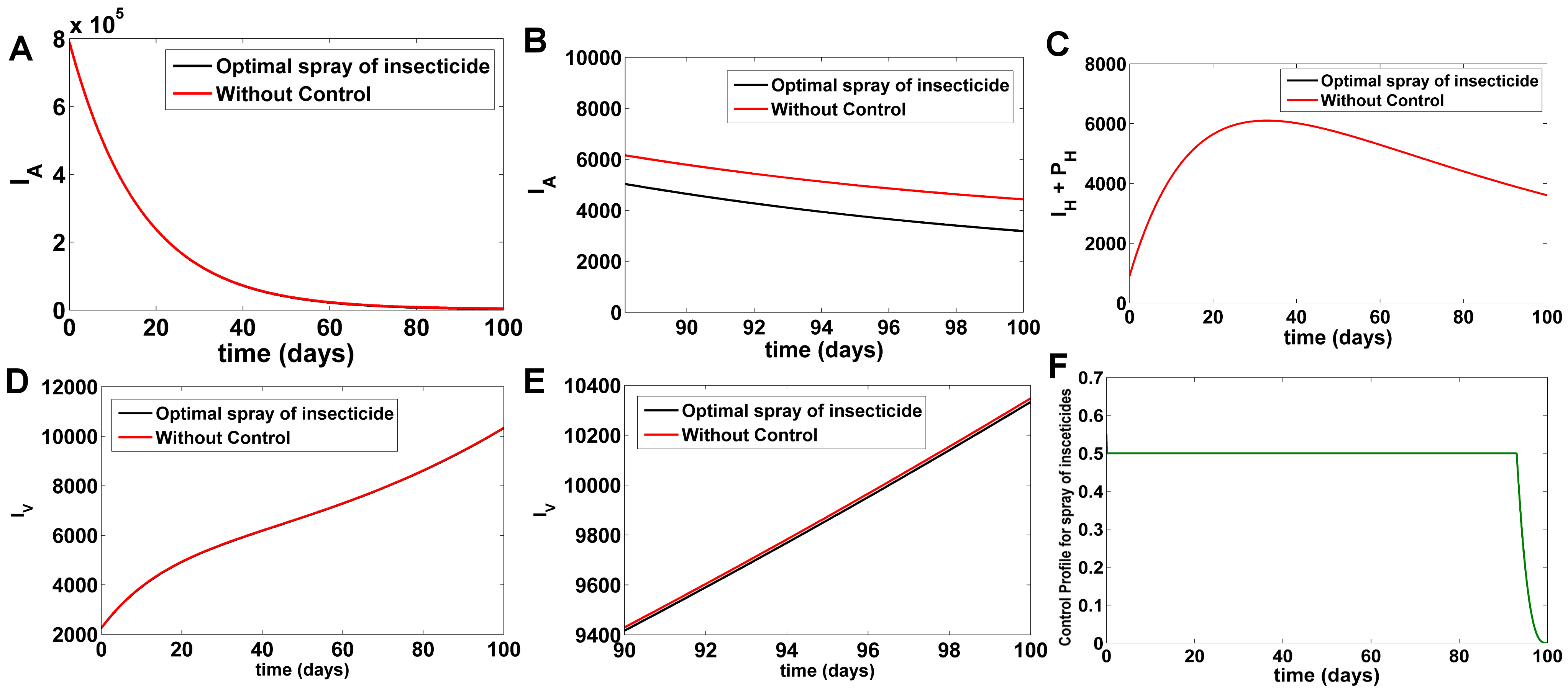

Supplement: S5 Fig — A) Number of asymptomatic KA individuals, B) S5.A Fig (magnified, t = 90–100 days), C) Number of symptomatic KA and PKDL infected individuals, D) Number of infected vectors, E) Control profile for optimal spray of insecticide policy. (TIF) [file pone.0172465.s006.tif]

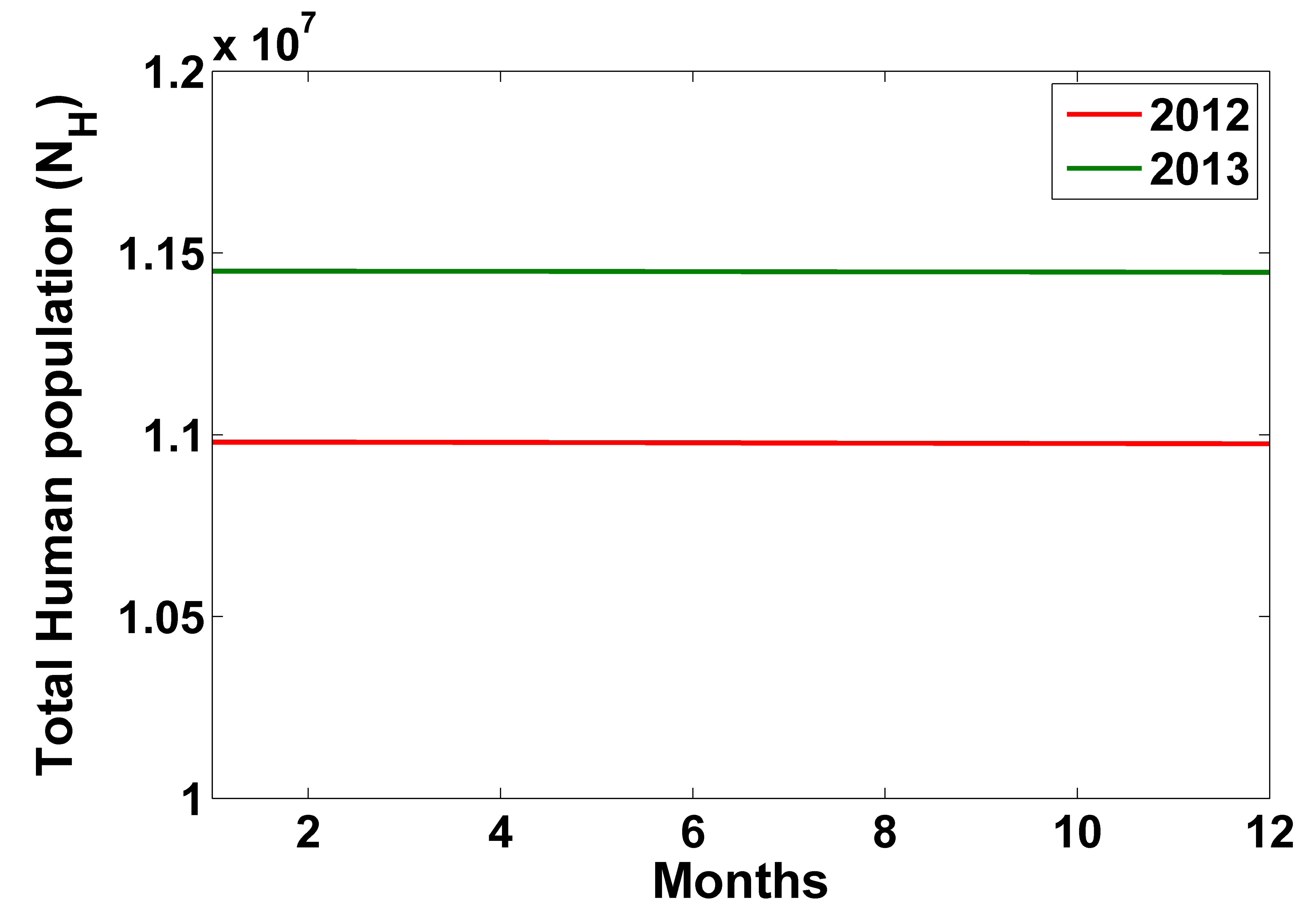

Supplement: S6 Fig — (TIF) [file pone.0172465.s007.tif]
